# *Salmonella enterica* serovar Typhimurium remodels mitochondrial dynamics of macrophages via the T3SS effector SipA to promote intracellular proliferation

**DOI:** 10.1080/19490976.2024.2316932

**Published:** 2024-02-14

**Authors:** Xingmei Liu, Yutao Liu, Xinyu Zhao, Xueping Li, Ting Yao, Ruiying Liu, Qian Wang, Qiushi Wang, Dan Li, Xintong Chen, Bin Liu, Lu Feng

**Affiliations:** a National Key Laboratory of Intelligent Tracking and Forecasting for Infectious Diseases, TEDA Institute of Biological Sciences and Biotechnology, Nankai University, Tianjin, China; b Key Laboratory of Molecular Microbiology and Technology, Nankai University, Tianjin, China; c Nankai International Advanced Research Institute, Nankai University Shenzhen, Shenzhen, China

**Keywords:** Effectors, mitochondrial fragmentation, *salmonella* typhimurium, SipA, pathogenicity

## Abstract

Mitochondrial dynamics are critical in cellular energy production, metabolism, apoptosis, and immune responses. Pathogenic bacteria have evolved sophisticated mechanisms to manipulate host cells’ mitochondrial functions, facilitating their proliferation and dissemination. *Salmonella enterica* serovar Typhimurium (*S*. Tm), an intracellular foodborne pathogen, causes diarrhea and exploits host macrophages for survival and replication. However, *S*. Tm-associated mitochondrial dynamics during macrophage infection remain poorly understood. In this study, we showed that within macrophages, *S*. Tm remodeled mitochondrial fragmentation to facilitate intracellular proliferation mediated by *Salmonella* invasion protein A (SipA), a type III secretion system effector encoded by *Salmonella* pathogenicity island 1. SipA directly targeted mitochondria via its N-terminal mitochondrial targeting sequence, preventing excessive fragmentation and the associated increase in mitochondrial reactive oxygen species, loss of mitochondrial membrane potential, and release of mitochondrial DNA and cytochrome *c* into the cytosol. Macrophage replication assays and animal experiments showed that mitochondria and SipA interact to facilitate intracellular replication and pathogenicity of *S*. Tm. Furthermore, we showed that SipA delayed mitochondrial fragmentation by indirectly inhibiting the recruitment of cytosolic dynamin-related protein 1, which mediates mitochondrial fragmentation. This study revealed a novel mechanism through which *S*. Tm manipulates host mitochondrial dynamics, providing insights into the molecular interplay that facilitates *S*. Tm adaptation within host macrophages.

## Introduction

Mitochondria play crucial roles in various cellular processes, including energy production, cellular metabolism, apoptosis regulation, and innate immunity.^1^ The roles of mitochondria in these cellular processes are closely interconnected with mitochondrial physiological functions, such as the maintenance of mitochondrial membrane potential (∆Ψ_m_), modulation of mitochondrial reactive oxygen species (mtROS) production, and limitation of mtDNA and mitochondrial cytochrome *c* release, which are strongly influenced by the highly dynamic nature of the mitochondria and further alter the mitochondria-mediated signaling pathways.^2^ For example, mitochondrial outer membrane permeabilization (MOMP) results in cytochrome *c* release from the mitochondria into the cytosol, activating caspases to induce apoptosis.^3^ The cytoplasmic release of mtDNA from dysfunctional mitochondria directly triggers antiviral and inflammatory responses as damage-associated molecular patterns (DAMPS).^4^ Additionally, mtROS production plays a role in coordinating innate immune responses, including regulating inflammatory cytokine secretion, antimicrobial responses, and immune cell activation.^4^ Mitochondrial dynamics are determined by the equilibrium of fission (fragmentation), resulting in a separated fragmented/rounded morphology, and fusion (elongation), causing an elongated and interconnected tubular network morphology, which tightly regulates mitochondrial shape, length, and number.^5^ Maintaining a balance between mitochondrial fragmentation and elongation is pivotal for maintaining mitochondrial function and cell fate. The imbalance between the opposing processes of mitochondrial fragmentation and elongation causes dysfunction and further affects mitochondria-mediated cellular signaling pathways. For example, bacterial infection can induce mitochondrial cell death pathways through classical mechanisms involving mitochondrial membrane permeabilization and ∆Ψ_m_ disruption.^6^ The subsequent release of mitochondrial cytochrome *c* into the host cell cytoplasm triggers apoptosome and associated caspase activity.^6^ The balance between mitochondrial fragmentation and elongation can be disrupted by various environmental stimuli, including bacterial infection, leading to excessive fragmentation that may destroy cellular ATP biogenesis and cause mitochondrial dysfunction. Recruitment of the cytosolic GTPase-protein dynamin-related protein 1 (Drp1), also called dynamin 1-like protein (DNM1L), from the cytosol to the mitochondrial surface primarily induces mitochondrial fragmentation.^7^ Increased Drp1 recruitment to mitochondria results in substantial mitochondrial fragmentation, decreasing ΔΨ_m_, increasing mtROS production, releasing cytochrome *c*, and eventually leading to apoptosis.^8–10^

Some pathogens have evolved mechanisms to modulate mitochondrial dynamics and interfere with essential mitochondrial functions that promote their pathogenesis.^11^ Pathogens primarily influence mitochondrial function by secreting effectors that specifically target and localize to the mitochondria.^12^ Multiple bacterial effectors induce or inhibit mitochondrial fragmentation to regulate different cellular responses. For example, the effector MitF secreted by *Legionella pneumophila* activates Ran GTPase, a small GTPase involved in various nuclear processes, and triggers Drp1 accumulation in the mitochondria to induce mitochondrial fragmentation, impairing oxidative phosphorylation (OXPHOS) and causing a Warburg-like metabolism that promotes a suitable environment for bacterial replication.^13^ *Vibrio cholera* injects its type III secretion system (T3SS) effector VopE into the host cell, which binds to mitochondrial Rho GTPases Miro1 and Miro2 and stimulates their GTPase activity, preventing Mitofusin1 (Mfn1)-induced mitochondrial fusion.^14^ VacA from *Helicobacter pylori* targets the mitochondrial inner membrane and induces mitochondrial network fragmentation, accompanied by ΔΨ_m_ loss and MOMP induction, to release cytochrome *c* and initiate apoptosis for bacterial persistence in the gastric environment.^15^ In contrast to mitochondrial fragmentation induced by several effectors, an effector secreted by *Chlamydia trachomatis* into the cytosol of host cells inhibits Drp1 recruitment to the mitochondria, stabilizes the mitochondrial fusion network, and degrades proapoptotic proteins, ultimately suppressing apoptosis for bacterial intracellular survival.^16–18^

*S*. Tm, an important gram-negative intracellular vacuolar bacterium, can cause gastroenteritis in humans and typhoid-like systemic illnesses in mice.^19^ *S*. Tm is acquired from contaminated food and water via the fecal – oral route. Once ingested, *S*. Tm manages to invade the intestinal barrier, penetrate intestinal epithelial cells, and is engulfed by macrophages, representing a crucial colonization niche for bacterial proliferation. After entry into host macrophages, *S*. Tm resides and replicates in customized *Salmonella*-containing vacuoles (SCVs), which are specialized membrane-bound compartments. To ensure intracellular survival, *S*. Tm encodes two distinct T3SS: *Salmonella* pathogenicity island 1 (SPI1) T3SS (T3SS1) and SPI2 T3SS (T3SS2), which inject multiple bacterial proteins into the host cytoplasm to subvert multiple host functions.^20^ *S*. Tm pathogenesis is highly dependent on two distinct T3SS and their secreted effectors. SPI1 effectors are essential for the initial invasion of *S*. Tm into epithelial cells by inducing membrane ruffling and cytoskeletal rearrangement,^21^ whereas some SPI1 effectors also contribute to intracellular replication in macrophages.^22–25^ SPI2 effectors primarily promote intracellular replication in epithelial cells and macrophages.^26^ Of the 47 reported SPI1 and SPI2 effectors, only SopB and SipB secreted by T3SS1 interact with the mitochondria. SopB interacts with mitochondria during invasion into epithelial cells and decreases mtROS production by retarding TRAF6 recruitment to the mitochondria, resulting in the inhibition of pro-apoptotic protein BAX translocation to the mitochondria and mitochondrial cytochrome *c* release, thus preventing host cell apoptosis for bacterial replication.^27^ SipB targets mitochondria in macrophages and disrupts mitochondrial morphology to induce the formation of autophagosome-like multilamellar structures, which cause autophagy-mediated cell death, thus avoiding host defense.^28^ However, the global effects and mechanisms underlying the successful infection of macrophages by *S*. Tm through the modulation of mitochondrial dynamics and function remain poorly understood.

SipA, secreted by T3SS1, is a well-characterized *Salmonella* virulence protein with multiple functions that facilitate both epithelial invasion and intracellular replication. During the invasion of epithelial cells, SipA binds to host actin, contributing to the cytoskeletal rearrangements that play a crucial role in the entry of *S*. Tm into epithelial cells.^29,30^ Furthermore, SipA causes transepithelial migration of polymorphonuclear leukocytes (PMNs) after attachment to epithelial cells.^31^ During *Salmonella* replication in macrophages, SipA is exposed on the cytoplasmic surface of SCV, facilitating SCV maturation and intracellular replication.^24^ However, whether SipA interacts with organelles in membrane structures, such as mitochondria or Golgi complexes, is unknown.

In this study, we analyzed the impact of mitochondrial dynamics during *S*. Tm infection in macrophages. Immunofluorescence and intracellular replication assays demonstrated that *S*. Tm delayed LPS-induced mitochondrial fragmentation during infection, thereby promoting intracellular replication in macrophages. Subsequently, we analyzed the colocalization of all 47 known effectors encoded by SPI1 and SPI2 of *S*. Tm with mitochondria. Among these effectors, SipA exhibited one of the highest Pearson’s correlation coefficients with mitochondria, as confirmed by immunofluorescence analysis. In addition, the immunofluorescence, qPCR, and immunoblotting assays showed that SipA prevented mtROS excessive increases, ΔΨ_m_ losses, and mtDNA and cytochrome *c* releases from mitochondria into the cytosol by targeting mitochondria to inhibit mitochondrial excessive fragmentation. Furthermore, macrophage replication assays and animal experiments showed that SipA-inhibited mitochondrial dysfunction contributes to intracellular replication and pathogenicity of *S*. Tm. Finally, using immunoblotting and immunoprecipitation, we showed that SipA inhibited the recruitment of Drp1 to the mitochondria through an indirect interaction. Overall, our findings reveal the mechanism by which SipA alters the mitochondrial dynamics of macrophages to facilitate the intracellular replication of *S*. Tm.

## Results

### S. Tm prevents host mitochondrial fragmentation to promote intracellular replication

**Figure 1.**
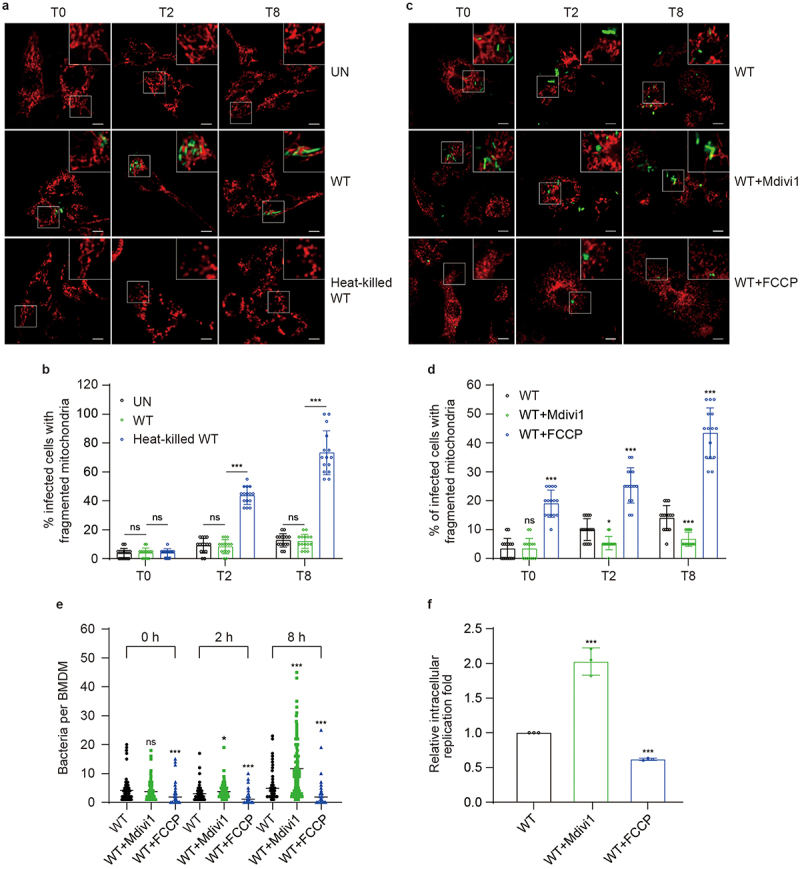
*S*. Tm prevents host mitochondrial fragmentation to promote intracellular replication.

To investigate whether *S*. Tm infection affects mitochondrial dynamics in macrophages, we examined the mitochondrial morphology of bone marrow-derived macrophages (BMDMs) infected with or without wild-type *S*. Tm (WT) at 2 and 8 h post-infection (p.i.) by immunofluorescence using laser scanning confocal microscopy. The WT-infected and uninfected (UN) BMDMs shared similar mitochondrial morphology, with no significant difference in the proportion of cells with fragmented mitochondria (Figure 1(a,b)), indicating that *S*. Tm infection does not affect mitochondrial dynamics during infection. However, the proportion of cells with fragmented mitochondria increased by 5.24- and 6.11-fold at 2 and 8 h p.i. in BMDMs infected with the heat-killed WT, respectively, compared with WT-infected BMDMs (Figure 1(a,b)). This effect of heat-killed WT on mitochondria dynamics is likely due to lipopolysaccharides (LPS), which is known to induce mitochondrial fragmentation.^32^ These results indicate that *S*. Tm impedes LPS-induced mitochondrial fragmentation in BMDMs, which is important for the intracellular survival of *S*. Tm.

(a) Representative immunofluorescence images of mitochondrial morphology in BMDMs infected with GFP-expressing WT and heat-killed WT at 0, 2, and 8 h p.i. Mitochondria, red; WT, green. Scale bars, 5 µm. (b) The proportion of cells with fragmented mitochondria after being infected with GFP-expressing WT and heat-killed WT at 0, 2, and 8 h p.i. (c) Representative immunofluorescence images of mitochondrial morphology in Mdivi1- or FCCP-treated BMDMs infected with GFP-expressing WT at 0, 2, and 8 h p.i. Mitochondria, red; WT, green. Scale bars, 10 µm. (d) The proportion of cells with fragmented mitochondria after pretreatment with Mdivi1 or FCCP followed by infection with GFP-expressing WT at 0, 2, and 8 h p.i. (e) Immunofluorescence analysis of the intracellular bacteria per BMDM after pretreatment with Mdivi1 or FCCP followed by infection with GFP-expressing WT at 0, 2, and 8 h p.i. (f) Relative replication fold of WT in BMDMs pretreated with Mdivi1 or FCCP. The WT (b, d – f) served as the control. UN, uninfected. Data are presented as mean ± SD (*n* = 15 fields (b, d), *n* = 100 cells (e), or *n* = 3 independent experiments (f)). Significant differences were assessed using two-way ANOVA (b, d, e, f). Error bars represent SD. *P < .05, ***P < .001; ns, no significant difference.

Mdivi1 and FCCP are used as inhibitors and activators of mitochondrial fragmentation.^33,34^ To determine the impact of mitochondrial morphology on the intracellular replication of *S*. Tm, BMDMs were pretreated with Mdivi1 or FCCP for 4 h (Fig. S1a), and untreated BMDMs were infected with *S*. Tm. The proportion of cells with fragmented mitochondria in FCCP-treated BMDMs was 2.53- and 3.10-fold higher than that in untreated BMDMs at 2 and 8 h p.i. (Figure 1(c,d)), whereas that with fragmented mitochondria in Mdivi1-treated BMDMs was 1.88- and 2.10-fold lower than that in untreated BMDMs at 2 and 8 h p.i. (Figure 1(c,d)). The average number of bacteria in FCCP-treated BMDM decreased by 2.69- and 2.68-fold whereas that in Mdivi1-treated BMDM increased by 1.23- and 2.37-fold at 2 and 8 h p.i. compared with that in untreated BMDM (Figure 1e). These data indicate a negative correlation between the proportion of cells with fragmented mitochondria and *S*. Tm replication in BMDMs. The effect of mitochondrial morphology on *S*. Tm replication was confirmed using intracellular replication assays. The relative replication of *S*. Tm in FCCP-treated BMDMs was 1.62-fold lower, whereas that in Mdivi1-treated BMDMs was 2.03-fold higher than that in untreated BMDMs (Figure 1f). These results indicate that inhibition of mitochondrial fragmentation enhances the intracellular replication of *S*. Tm within BMDMs.

Together, the above results indicate that *S*. Tm effectively inhibits LPS-induced host mitochondrial fragmentation in BMDMs, which is critical for intracellular replication of *S*. Tm.

### SipA is required to prevent mitochondrial fragmentation during infection

**Figure 2.**
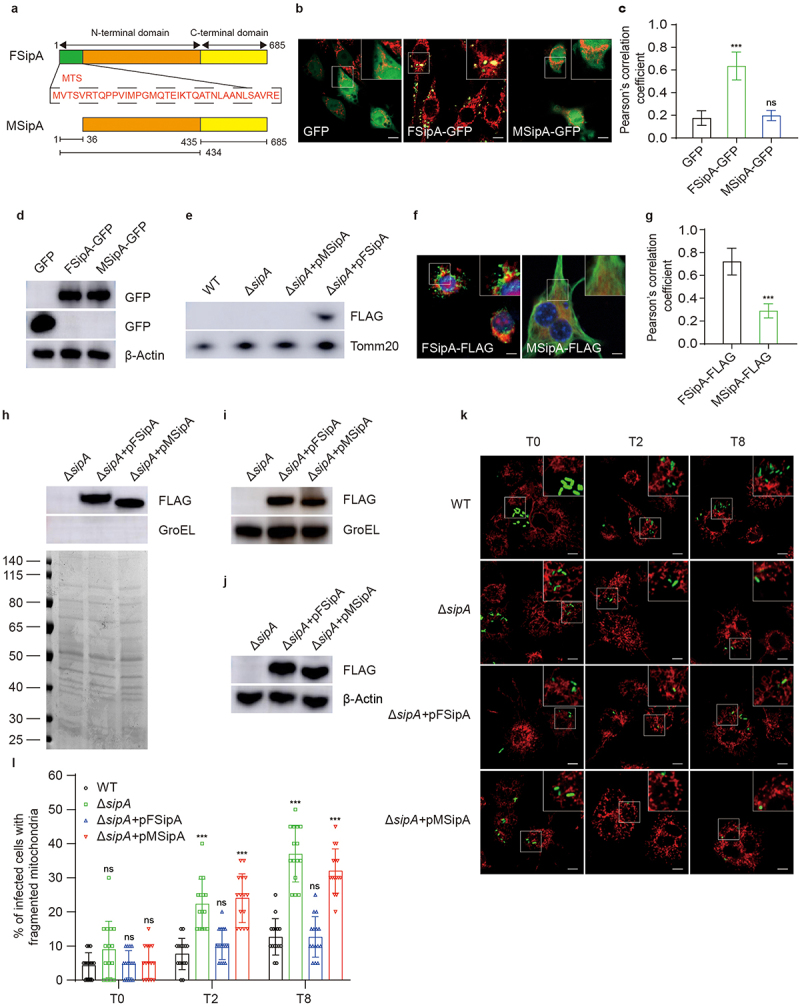
SipA is required to prevent mitochondrial fragmentation during infection.

Next, we investigated the mechanism underlying *S*. Tm’s inhibition of host mitochondrial fragmentation during infection. Because pathogens can secrete effectors targeting mitochondria to modulate the mitochondrial dynamics of host cells, we evaluated the colocalization of 47 known effectors encoded by SPI1 and SPI2 of *S*. Tm with mitochondria. First, we constructed eukaryotic expression vectors for 47 effector-GFP fusion proteins and transfected them into RAW264.7 macrophages. Except for 13 effectors that could not be transfected into RAW264.7, we analyzed the colocalization of the other 34 effectors with mitochondria by immunofluorescence. The Pearson’s correlation coefficient for the colocalization of the 11 effectors with mitochondria, including SipB and SopA, which have been previously shown to target mitochondria,^28,35^ was significantly higher than that observed between GFP expressed by the empty vector and mitochondria (Fig. S2a, b). Among these 11 effector proteins, SipA significantly colocalized with the mitochondria, with a maximum Pearson’s correlation coefficient of 0.64 (Figure 2c), indicating that SipA specifically localizes to the mitochondria. We hypothesized that SipA plays a major role in preventing mitochondrial fragmentation in macrophages.

(a) Domain architectures of *S*. Tm 14028s FSipA and MSipA. (b) Representative immunofluorescence images of Raw264.7 macrophages transfected with pCMV-C-GFP, pCMV-FSipA-GFP, or pCMV-MSipA-GFP plasmids after 24 h. Mitochondria, red; the indicated proteins, green. Scale bars, 10 µm. (c) Pearson’s correlation coefficient for the indicated protein and mitochondria colocalization after 24 h of transfection. (d) Expression levels of SipA in Raw264.7 macrophages transfected with pCMV-C-GFP, pCMV-FSipA-GFP, or pCMV-MSipA-GFP plasmids for 24 h followed by immunoblotting with antibody against GFP tag. (e) The analysis of colocalization for SipA and mitochondria by immunoblotting using antibody against the FLAG tag. (f) Representative immunofluorescence images of BMDMs infected with the Δ*sipA+*pFSipA or Δ*sipA*+pMSipA at 8 h p.i. FSipA and MSipA were tagged with FLAG. Mitochondria, red; the indicated protein, green; nucleus, blue. Scale bars, 5 µm. (g) Pearson’s correlation coefficient for the indicated protein and mitochondria colocalization at 8 h p.i. (h) Detection of SipA secreted by Δ*sipA*, Δ*sipA+*pFSipA and Δ*sipA+*pMSipA under T3SS inducing conditions in bacterial culture supernatants. Total proteins were quantified by SDS-PAGE stained with Coomassie blue staining. Protein SipA was detected by immunoblotting using antibody against the FLAG tag. (i) Detection of SipA in bacterial cell lysates from Δ*sipA*, Δ*sipA+*pFSipA and Δ*sipA+*pMSipA by immunoblotting using antibody against the FLAG tag. (j) Detection of SipA secreted in BMDMs infected with Δ*sipA*, Δ*sipA+*pFSipA and Δ*sipA+*pMSipA by immunoblotting with an antibody against the FLAG tag. (k) Representative immunofluorescence images of mitochondrial morphology in BMDMs infected with GFP-expressing WT, Δ*sipA*, Δ*sipA+*pFSipA, or Δ*sipA+*pMSipA at 0, 2, and 8 h p.i. Mitochondria, red; *S*. Tm, green. Scale bars, 10 µm. (l) The proportion of cells with fragmented mitochondria after being infected with GFP-expressing WT, Δ*sipA*, Δ*sipA+*pFSipA, or Δ*sipA+*pMSipA at 0, 2, and 8 h p.i. The GFP (c), FSipA (g), or WT (l) served as controls. FSipA, full-length SipA; MSipA, MTS-deleted SipA. β-Actin, cytosolic control; Tomm20, mitochondrial control; GroEL, bacterial control. Data are presented as mean ± SD (*n* = 30 fields (c, g), *n* = 15 fields (l)). Significant differences were assessed by two-tailed unpaired Student’s *t*-test (g) or two-way ANOVA (c, l). Error bars represent SD. ***P < .001,; ns, no significant difference.

Positively charged mitochondrial targeting sequence (MTS) is a short peptide essential for protein transport to the mitochondria. Analysis using MitoProt (http://ihg.gsf.de/ihg/mitoprot.html) revealed a potential N-terminal MTS in SipA (Figure 2(a)). SipA likely targets the mitochondria via this N-terminal MTS. To test this hypothesis, MTS-deleted SipA (MSipA) eukaryotic expression vectors were constructed and transfected into RAW264.7 macrophages. Immunofluorescence analysis showed that the deletion of the MTS in SipA resulted in its failure to localize to the mitochondria (Figure 2(b)). Further colocalization analysis demonstrated that MSipA could not localize to the mitochondria (Figure 2(c)), indicating that the MTS is crucial for the mitochondrial localization of SipA. Meanwhile, the expression level of full-length SipA (FSipA) or MSipA were evaluated by immunoblotting. The results showed the expression level of MSipA was comparable to that of FSipA (Figure 2(d) and S3(a)). To further validate the above conclusion, BMDMs were infected by WT, Δ*sipA*, Δ*sipA* complemented with pTr-FSipA-FLAG (Δ*sipA+*pFSipA), and Δ*sipA* complemented with pTr-MSipA-FLAG (Δ*sipA+*pMSipA) at 8 h p.i. The mitochondria in these infected cells were isolated, and the localization of SipA on the mitochondria was detected by immunoblotting. SipA was localized to the mitochondria in Δ*sipA+*pFSipA-infected BMDMs (Figure 2(e)), whereas SipA could not be detected in mitochondria from WT-, Δ*sipA*-, or Δ*sipA+*pMSipA-infected BMDMs (Figure 2(e)). Furthermore, SipA localization in the mitochondria of BMDMs infected with Δ*sipA+*pFSipA or Δ*sipA+*pMSipA was analyzed by immunofluorescence using confocal laser scanning microscopy. Consistent with these results, FSipA, but not MSipA, was localized to the mitochondria at 8 h p.i. (Figure 2(f,g)). Considering that the majority of T3SS effectors possess N-terminal secretion signals which is essential for their translocation, the immunoblotting was performed to determine the secretion and expression of SipA from Δ*sipA*, Δ*sipA*+pFSipA and Δ*sipA*+pMSipA under T3SS inducing conditions for 3 h. The result showed MSipA can still be detected in the bacterial culture supernatants (Figure 2h, S3b) and stably expressed in the bacterial cells (Figure 2(i) and S3(c)), indicating the absence of N-terminal MTS doesn’t affect SipA secretion and expression in vitro. Furthermore, the immunoblotting were also performed to detect the secretion of SipA in cell lysate supernatants of BMDMs infected with Δ*sipA*, Δ*sipA+*pFSipA and Δ*sipA+*pMSipA at 8 h p.i. The results showed a comparable protein level for FSipA and MSipA was observed in cell lysate supernatants (Figure 2(j) and S3(d)), indicating secretion of SipA does not depend on its MTS. These data indicate that the N-terminal MTS is required for SipA to target the host mitochondria.

We further investigated whether SipA was required to prevent mitochondrial fragmentation during infection. First, BMDMs were infected by WT, Δ*sipA*, or Δ*sipA+*pFSipA. The mitochondrial morphology of infected cells was determined by immunofluorescence and observed using confocal laser scanning microscopy. The proportion of cells with fragmented mitochondria in Δ*sipA*-infected BMDMs was 2.91- and 2.92-fold higher than that in WT-infected BMDMs at 2 and 8 h p.i., respectively (Figure 2(k,l)). However, the Δ*sipA+*pFSipA-infected BMDMs exhibited a similar mitochondrial fragmentation ratio to that of WT-infected BMDMs (Figure 2(k,l)). These data indicated that SipA prevents mitochondrial fragmentation during infection. To confirm that targeting mitochondria via the N-terminal MTS is essential for the function of SipA in preventing mitochondrial fragmentation, we infected BMDMs with Δ*sipA+*pMSipA, and the mitochondrial morphology of the infected cells was detected by immunofluorescence and monitored by confocal laser scanning microscopy. The results showed that the proportion of cells with fragmented mitochondria in Δ*sipA+*pMSipA-infected BMDMs exhibited no significant difference compared with that in Δ*sipA-*infected BMDMs but was significantly higher than that in WT-infected BMDMs and Δ*sipA+*pFSipA-infected BMDMs at 2 and 8 h p.i. (Figure 2(k,l)). These data indicate that MTS plays a key role in SipA-mediated inhibition of mitochondrial fragmentation. In conclusion, these data demonstrate that *S*. Tm secretes SipA during infection to prevent mitochondrial fragmentation by directly targeting mitochondria, which is mediated by the N-terminal MTS of SipA.

### Lack of SipA increases mitochondrial dysfunction

**Figure 3.**
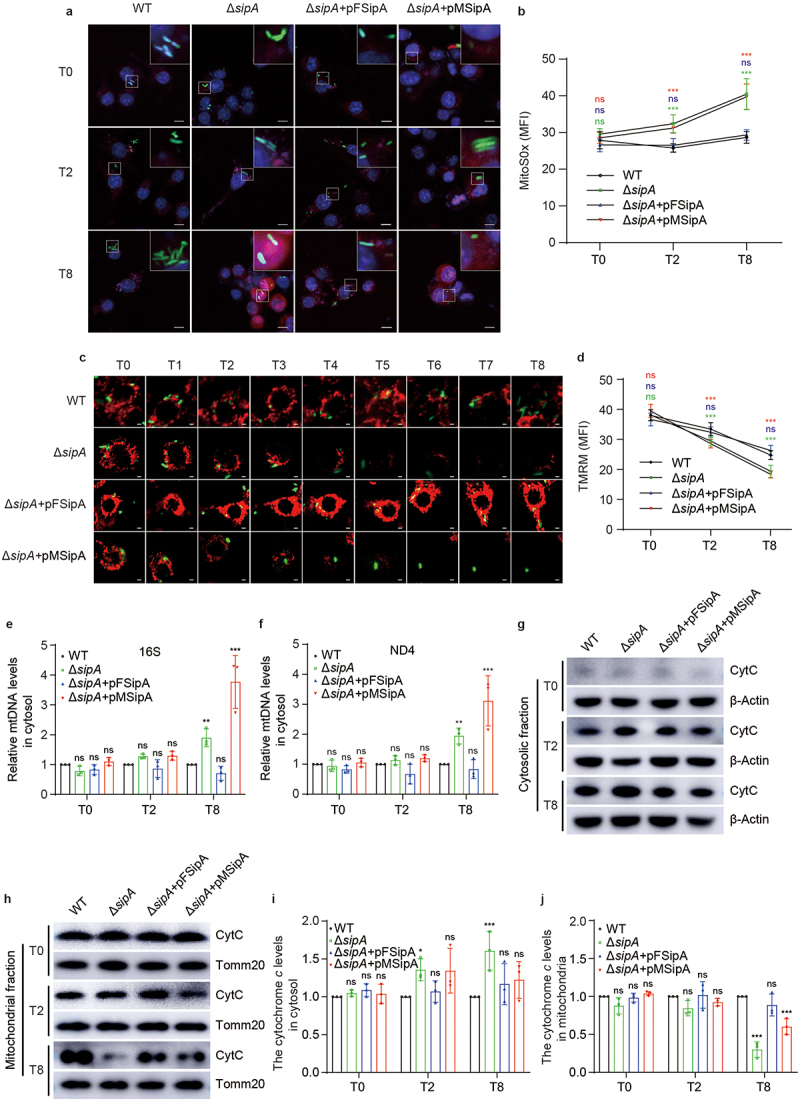
Lack of *sipA* increases mitochondrial dysfunction.

Above results showed that SipA targeted mitochondria to prevent mitochondrial fragmentation. As mitochondrial fragmentation leads to mitochondrial dysfunction, we hypothesized that SipA prevents mitochondrial dysfunction during infection. Mitochondrial fragmentation commonly leads to excessive production of mtROS.^36^ We first investigated whether SipA plays a role in the attenuation of mtROS production. The BMDMs were infected with WT, Δ*sipA*, or Δ*sipA+*pFSipA and stained with MitoSOX Red to detect mtROS levels. The immunofluorescence results showed that at 2 and 8 h p.i., the mean fluorescence intensity (MFI) of mtROS in Δ*sipA-*infected BMDMs was significantly higher than that in WT-infected BMDMs (Figure 3(a,b)), whereas the Δ*sipA+*pFSipA-infected BMDMs exhibited a similar MFI of mtROS to that of WT-infected BMDMs (Figure 3(a,b)), indicating that SipA inhibits mtROS production.

(a) Representative immunofluorescence images of mtROS in BMDMs infected with GFP-expressing WT, Δ*sipA*, Δ*sipA+*pFSipA, or Δ*sipA+*pMSipA at 0, 2, and 8 h p.i. mtROS, red; *S*. Tm, green; nucleus, blue. Scale bars, 10 µm. (b) MitoSOX fluorescence intensity analysis of mtROS in BMDMs infected with GFP-expressing WT, Δ*sipA*, Δ*sipA+*pFSipA, or Δ*sipA+*pMSipA at 0, 2, and 8 h p.i. (c) The time-lapse photography of ΔΨ_m_ in BMDMs infected with GFP-expressing WT, Δ*sipA*, Δ*sipA+*pFSipA, or Δ*sipA+*pMSipA during 8 h p.i. ΔΨ_m_, red; *S*. Tm, green. Scale bars, 2 µm. (d) TMRM fluorescence intensity analysis of ΔΨ_m_ in BMDMs infected with GFP-expressing WT, Δ*sipA*, Δ*sipA+*pFSipA, or Δ*sipA+*pMSipA at 0, 2, and 8 h p.i. (e, f) The qPCR analysis of cytosolic mtDNA levels in BMDMs infected with WT, Δ*sipA*, Δ*sipA+*pFSipA, or Δ*sipA+*pMSipA at 0, 2, and 8 h p.i. (g, h) Representative immunoblotting image of cytosolic (g) and mitochondrial (h) cytochrome *c* levels in BMDMs infected with WT, Δ*sipA*, Δ*sipA+*pFSipA, or Δ*sipA+*pMSipA at 0, 2, and 8 h p.i. (i, j) Immunoblotting analysis of cytosolic (i) and mitochondrial (j) cytochrome *c* levels. The WT served as the control (b, d – f, i, j). CytC, cytochrome *c*. β-Actin, cytosolic control; Tomm20, mitochondrial control. Data are presented as mean ± SD (*n* = 15 fields (b, d) or *n* = 3 independent experiments (e, f, i, j)). Significant differences were assessed using two-way ANOVA (b, d – f, i, j). Error bars represent SD. *P < .05, **P < .01, ***P < .001; ns, no significant difference.

The excessive production of mtROS can further induce the mitochondrial permeability transition,^37^ resulting in the dissipation of ΔΨ_m_. Thus, we investigated whether SipA prevents the loss of ΔΨ_m_ during infection. The BMDMs were infected with WT, Δ*sipA*, or Δ*sipA+*pFSipA and stained with a ΔΨ_m_-dependent mitochondrial dye tetramethylrhodamine methyl ester (TMRM). The MFI of ΔΨ_m_ was detected by confocal laser scanning microscopy. The time-lapse confocal results revealed a gradual decline of ΔΨ_m_ in BMDMs infected with WT, Δ*sipA*, or Δ*sipA+*pFSipA over 8 h p.i. (Figure 3(c)). Furthermore, the MFI analysis results of ΔΨ_m_ showed that at 2 and 8 h p.i., the ΔΨ_m_ of BMDMs infected with Δ*sipA* was significantly lower than that in BMDMs infected with WT (Figure 3(d)), whereas there was no significant difference in MFI of ΔΨ_m_ between Δ*sipA+*pFSipA-infected and WT-infected BMDMs (Figure 3(d)). These data indicate that SipA can repress the loss of ΔΨ_m_.

The mitochondrial permeability transition leads to the release of mtDNA and cytochrome *c* from mitochondria into the cytosol. We subsequently assessed the effect of SipA on the translocation of mtDNA from mitochondria into the cytosol. The BMDMs were infected with WT, Δ*sipA*, or Δ*sipA+*pFSipA, and the isolated cytosolic mtDNA levels were quantified using qPCR. Δ*sipA* triggered a significantly higher release of mtDNA from mitochondria than WT at 8 h p.i. (Figure 3(e,f)), whereas Δ*sipA+*pFSipA induced a similar level of mtDNA release compared with WT (Figure 3(e,f)). This indicates that SipA can inhibit the release of mtDNA from the mitochondria into the cytosol during *S*. Tm infection.

To investigate the influence exerted by SipA on the translocation of cytochrome *c* from mitochondria into the cytosol, the BMDMs were infected with WT, Δ*sipA*, or Δ*sipA+*pFSipA and subjected to immunoblotting analysis for cytochrome *c* level measurements in mitochondria and cytosol. Δ*sipA* significantly increased cytochrome *c* levels in the cytosol by 1.36- and 1.61-fold compared with WT at 2 and 8 h p.i. (Figure 3(,i)). Meanwhile, compared with WT-infected BMDMs, cytochrome *c* levels decreased in the mitochondria of Δ*sipA*-infected BMDMs (Figure 3(h,j)). In contrast, cytochrome *c* levels in both the cytosol and mitochondria of Δ*sipA+*pFSipA-infected BMDMs exhibited no significant difference compared with that in WT-infected BMDMs at 2 and 8 h p.i. (Figure 3(g–j)). These results demonstrated that SipA efficiently suppressed the release of cytochrome *c* from the mitochondria into the cytosol during infection.

SipA could prevent mitochondrial fragmentation-induced mitochondrial dysfunction, including the production of mtROS, loss of ΔΨ_m_, and release of mtDNA and cytochrome *c* from mitochondria. We further investigated whether the MTS of SipA was indispensable for these effects. MtROS production, ΔΨ_m_ loss, and mtDNA and cytochrome *c* release from mitochondria in BMDMs infected with Δ*sipA+*pMsipA were comparable to those observed in Δ*sipA*-infected BMDMs (Figure 3(a–j)). These findings suggest that N-terminal MTS is essential for mitigating mitochondrial dysfunction by SipA.

### SipA enhances *S*. Tm intracellular replication by targeting mitochondria

**Figure 4.**
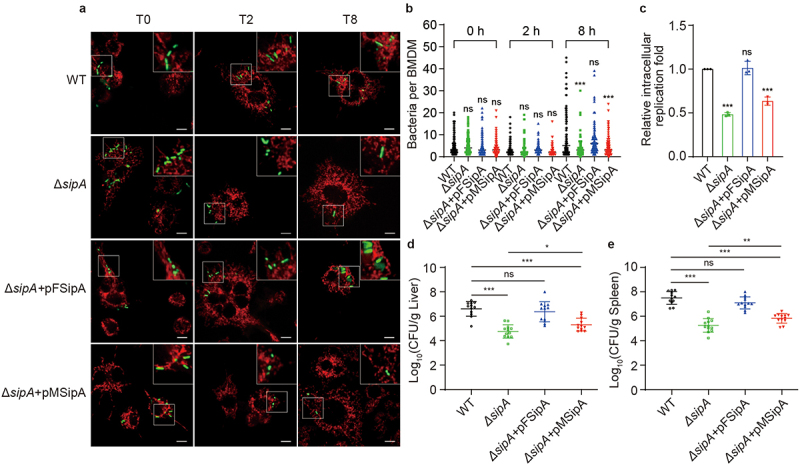
SipA enhances *S*. Tm intracellular replication by targeting mitochondria.

As the inhibition of host mitochondrial fragmentation is crucial for *S*. Tm intracellular replication in BMDMs, we next investigated whether SipA contributes to the intracellular replication of *S*. Tm by targeting the mitochondria. We quantified the bacterial load in BMDMs infected with WT, Δ*sipA*, Δ*sipA+*pFSipA or Δ*sipA+*pMSipA using confocal laser scanning microscopy. The average bacterial load in Δ*sipA-* and Δ*sipA+*pMsipA-infected BMDMs was significantly reduced compared with that in WT-infected BMDMs at 8 h p.i. (Figure 4(a,b)), while the average bacterial load showed no significant difference between Δ*sipA+*pFSipA- and WT-infected BMDMs (Figure 4(a,b)). This indicates that SipA-targeted mitochondria are essential for bacterial replication in BMDMs. To further confirm this conclusion, we conducted intracellular replication assays with WT, Δ*sipA*, Δ*sipA+*pFSipA or Δ*sipA+*pMSipA. The relative replication fold of Δ*sipA* and Δ*sipA+*pMSipA exhibited 2.07- and 1.58-fold decrease compared with that of WT at 8 h p.i. (Figure 4(c)). However, Δ*sipA+*pFSipA restored the replication ability of *S*. Tm to a level comparable to that of WT (Figure 4(c)). These data suggest that SipA contributes to the intracellular replication of *S*. Tm by targeting mitochondria.

(a) Representative immunofluorescence images of intracellular bacteria in BMDMs infected with GFP-expressing WT, Δ*sipA*, Δ*sipA+*pFSipA, or Δ*sipA+*pMSipA at 0, 2, and 8 h p.i. Mitochondria, red; *S*. Tm, green. Scale bars, 10 µm. (b) Immunofluorescence analysis of the intracellular bacteria per BMDM after infection with GFP-expressing WT, Δ*sipA*, Δ*sipA+*pFSipA, or Δ*sipA+*pMSipA at 0, 2, and 8 h p.i. (c) Relative replication fold of WT, Δ*sipA*, Δ*sipA+*pFSipA, or Δ*sipA+*pMSipA in BMDMs. (d, e) Liver and spleen bacterial burdens in BALB/c mice intraperitoneally infected with WT, Δ*sipA*, Δ*sipA+*pFSipA, or Δ*sipA+*pMSipA. The WT served as the control (b – e). Data are presented as mean ± SD (*n* = 100 cells (b), *n* = 3 independent experiments (c) or *n* = 12 mice per group (d, e)). Significant differences were assessed using two-tailed unpaired Student’s *t*-test (b, c) or Mann–Whitney U-test (d, e). Error bars represent SD. *P < .05, **P < .01, ***P < .001; ns, no significant difference.

In addition, mice infection assays were performed to confirm this conclusion *in vivo*. Mice were administered intraperitoneally with WT, Δ*sipA*, Δ*sipA+*pFSipA or Δ*sipA+*pMSipA. After 3 d, the liver and spleen were isolated to quantify the bacterial load. The results demonstrated a significant reduction in bacterial burdens in the liver and spleen of mice infected with Δ*sipA* compared to those infected with WT (Figure 4(d,e)). As expected, the Δ*sipA+*pFSipA was comparable to the WT in bacterial burdens in the liver and spleen of the infected mice (Figure 4(d,e)). However, the bacterial loads in the liver and spleen of Δ*sipA+*pMSipA-infected mice were only partially restored compared with that in WT-infected mice (Figure 4(d,e)), indicating that the interaction between SipA and mitochondria enhances the pathogenicity of *S*. Tm in animals. Because SipA plays numerous crucial roles during infection, the data suggest that the interaction between SipA and mitochondria is crucial for some of its effects that contribute to successful infection of *S*. Tm, but not all of them. Finally, we determined the growth curves of WT, Δ*sipA*, Δ*sipA+*pFSipA and Δ*sipA+*pMSipA in Luria – Bertani (LB) and RPMI 1640 medium. There was no growth defect among Δ*sipA*, Δ*sipA+*pFSipA and Δ*sipA+*pMSipA compared with WT in both LB and RPMI 1640 medium (Fig. S4a, b). These findings suggest that SipA enhances *in vitro* replication capacity and *in vivo* pathogenicity of *S*. Tm by selectively targeting mitochondria.

### SipA prevents the recruitment of Drp1 to host mitochondria

**Figure 5.**
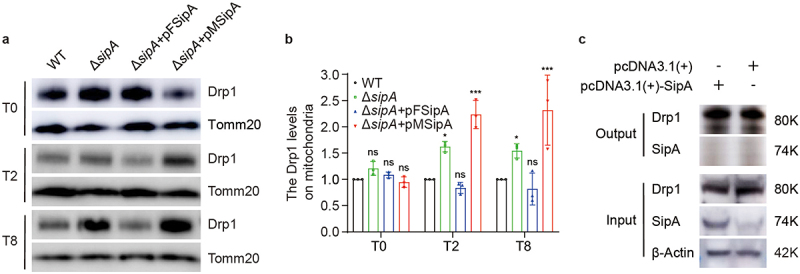
SipA prevents the recruitment of Drp1 to host mitochondria.

Mitochondrial fragmentation is closely regulated by Drp1 in eukaryotic cells.^38^ Thus, we investigated whether SipA inhibited mitochondrial fragmentation by attenuating Drp1 translocation to the mitochondria. The mitochondria of BMDMs infected with WT, Δ*sipA*, or Δ*sipA+*pFSipA were isolated, and the Drp1 protein levels recruited to mitochondria were detected by immunoblotting. At 2 and 8 h p.i., BMDMs infected with Δ*sipA* exhibited a 1.62- and 1.54-fold increase in Drp1 levels in mitochondria compared to WT-infected BMDMs (Figure 5(a,b)). Meanwhile, the protein levels of Drp1 in the mitochondria were similar between Δ*sipA+*pFSipA- and WT-infected BMDMs (Figure 5(a,b)). This indicates that a lack of *sipA* can promote the recruitment of Drp1 to the mitochondria during infection. Furthermore, we also analyzed the levels of Drp1 recruited to mitochondria in Δ*sipA+*pMSipA-infected BMDMs. The levels of Drp1 in the mitochondria of Δ*sipA+*pMSipA-infected BMDMs were significantly increased compared to that in WT-infected BMDMs (Figure 5(a,b)), indicating that SipA inhibits the recruitment of Drp1 to mitochondria by directly targeting mitochondria.

(a) Representative immunoblotting image of Drp1 level on mitochondria in BMDMs infected with WT, Δ*sipA*, Δ*sipA+*pFSipA, or Δ*sipA+*pMSipA at 0, 2, and 8 h p.i. (b) Immunoblotting analysis of Drp1 levels on mitochondria. (c) Immunoprecipitation of SipA and Drp1 in Raw264.7 macrophages. The WT served as the control (b). Tomm20, mitochondrial control; β-Actin, cytosolic control. Data are presented as mean ± SD (*n* = 3 independent experiments (b)). Significant differences were assessed using two-way ANOVA (b). Error bars represent SD. *P < .05, ***P < .001; ns, no significant difference.

We further investigated whether SipA directly interacts with Drp1 to inhibit its recruitment to mitochondria. The RAW264.7 macrophages were transfected with the SipA-expressing plasmid vector (pcDNA3.1(+)-SipA) or the pcDNA3.1(+) empty vector (control). Both cell lysates were subjected to immunoprecipitation using an anti-Drp1 antibody, followed by immunoblotting. Drp1 and SipA in RAW264.7 macrophages transfected with SipA-expressing plasmid could be detected in input samples. However, SipA was not detected in the corresponding output samples, which was consistent with the control cells, indicating that Drp1 did not coimmunoprecipitate with SipA (Figure 5(c) and S5(a)). These results demonstrate that SipA cannot directly bind to Drp1.

## Discussion

Pathogens have evolved sophisticated mechanisms to hijack mitochondria and modulate mitochondrial dynamics and function to promote survival and replication.^39^ However, the mitochondrial dynamics manipulated by *S*. Tm during infection remain poorly understood. In this study, we demonstrated that *S*. Tm inhibits mitochondrial fragmentation in macrophages. When engulfed by macrophages, *S*. Tm secretes the T3SS1 effector, SipA, which directly targets the mitochondria. Subsequently, by interfering with the recruitment of Drp1, SipA alters mitochondrial dynamics to reduce excessive fragmentation while avoiding excessive mtROS production, ΔΨ_m_ dissipation, and mtDNA and cytochrome *c* release from mitochondria. This benefits the intracellular replication of *S*. Tm and promotes its pathogenicity in the host.

Pathogens employ various strategies to manipulate mitochondrial processes to subvert the fate of the infected host. Numerous effectors secreted by bacteria influence mitochondrial dynamics and function during infection.^11^ At different infection stages, bacteria can influence mitochondrial dynamics through different effectors and mechanisms. For instance, in the early stages of enteropathogenic *Escherichia coli* (EPEC) infection, the bacterial effector EspZ interacts with mitochondrial fission protein 1 (FIS1) of host epithelial cells, a mitochondrial outer membrane protein that mediates mitochondrial fragmentation.^40^ This interaction protects the mitochondrial fusion network and enhances host cell viability, benefitting EPEC colonization. In contrast, at the late stage of infection, EPEC stimulates mitochondrial fragmentation via an EspH-dependent increase in FIS1 levels and causes a loss in ΔΨ_m_,^40^ leading to host cell death, which likely facilitates pathogen dispersal. In this study, at the early stages of *S*. Tm infection, SipA targets mitochondria to inhibit mitochondrial fragmentation and cell death, which benefits intracellular survival. The effector SseL of *S*. Tm induces cell death in macrophages at late time points after invasion.^41^ After entering macrophages, *S*. Tm may prevent host mitochondrial fragmentation to promote intracellular replication during the early stages of infection. However, at late time points during infection, it triggers macrophage death,^42^ which may benefit bacterial dissemination. Multiple effectors are probably engaged in the complex interactions between *S*. Tm and mitochondria during infection. Additionally, through colocalization analysis by immunofluorescence, we found that in addition to SipA and SipB, other nine effectors could also target the mitochondria of macrophages. These nine effectors likely modulate mitochondrial dynamics in macrophages via different mechanisms. However, the role of these effectors in regulating mitochondrial dynamics requires further investigation.

Multiple bacterial effectors are multitasking proteins that exert different roles during the interaction between host and pathogens. SipB induces the formation of multivesicular structures, including both mitochondrial and endoplasmic reticulum markers.^28^ In addition to regulating mitochondrial dynamics, SipA performs other functions during infection, such as polymorphonuclear leukocyte recruitment,^31^ actin cytoskeleton rearrangement^29,30^ and SCV targeting.^24^ When SipA is exposed to the cytoplasmic face of the SCV, the N-terminal domain of SipA specifically recruits syntaxin8 on SCVs, which is a soluble N-ethylmaleimide-sensitive factor attachment receptor (SNARE) protein involved in the regulation of phagosome maturation, to promote the fusion of SCV with early endosomes and inhibit the transport of SCV to lysosomes.^43^ These researches suggest that SipA is a multifunctional protein facilitating various biological processes to enhance *S*. Tm intracellular replication and pathogenicity. In this study, we revealed that SipA can directly target mitochondria and inhibit mitochondrial fragmentation. Moreover, we also validated that inhibiting mitochondrial fragmentation favors *S*. Tm intracellular replication, suggesting that SipA targeting mitochondria is one of strategy to promote *S*. Tm intracellular replication and pathogenicity. Although several mechanisms by which SipA enhances *Salmonella* intracellular replication and pathogenicity have been demonstrated, the comprehensive understanding of the interplay between these mechanisms in promoting intracellular replication and pathogenicity of *S*. Tm remains incomplete and warrants further investigation.

**Figure 6.**
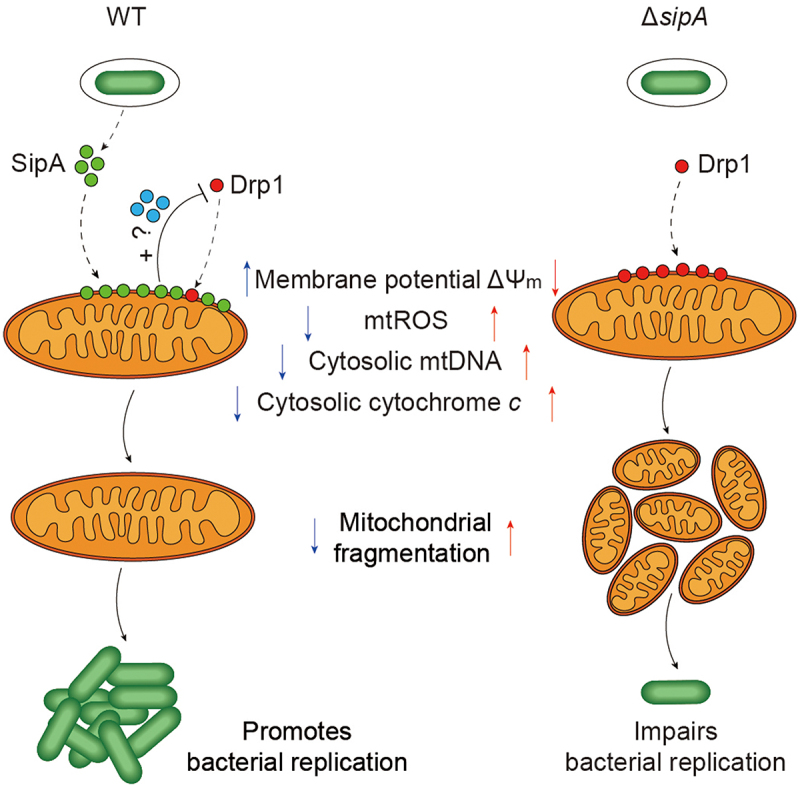
Model of *S*. Tm remodeling mitochondrial dynamics for its efficient replication.

Mitochondrial fragmentation and fusion play critical roles in maintaining functional mitochondria when cells experience metabolic or environmental stress. Mitochondrial fragmentation generates new organelles and facilitates quality control in growing and dividing cells, whereas fusion contributes to the response of mammalian cells to stress and energy demand.^44^ The Drp1-dependent pathway is the classical mechanism of mitochondrial fragmentation. For example, *Legionella pneumophila* effector Lpg1137 binds to and cleaves syntaxin17, a SNARE protein that regulates Drp1 activity during infection and induces mitochondrial fragmentation.^45^ In addition, *L. pneumophila* effector MitF triggers the activation of the Wiskott – Aldrich syndrome protein (WASP) and actin-related protein 2/3 complex (Arp2/3).^13^ Actin nucleation by WASP/Arp2/3 progressively facilitates Drp1-mediated mitochondrial fragmentation.^13^ In this study, *S*. Tm exploits the T3SS1 effector SipA to inhibit mitochondrial fragmentation in a Drp1-dependent manner (Figure 6). However, this study failed to identify the precise mechanism underlying the interaction between SipA and Drp1. SipA likely inhibits the recruitment of Drp1 to the mitochondria via unknown intermediates, which can be a subject for future studies (Figure 6).

When *S*. Tm enters the host cells, WT secretes effector SipA, which can target mitochondria and inhibit the recruitment of Drp1 to mitochondria through indirect interaction, thus inhibiting Drp1-mediated mitochondrial fragmentation for bacterial replication. In contrast, when it lacks effector SipA, Δ*sipA* fails to inhibit Drp1 recruitment to mitochondria and the following Drp1-mediated mitochondrial fragmentation, thus impairing bacterial replication.

## Materials and methods

### Mice

Female BALB/c mice (8 weeks old) were purchased from Beijing Vital River Laboratory Animal Technology Co., Ltd. (Beijing, China). All mice were housed in a specific pathogen-free environment and fed a standard mouse chow diet. All animal experiments adhered to the criteria approved by the Institutional Animal Care Committee of Nankai University (Tianjin, China).

### Bacterial strains and plasmids

The bacterial strains and plasmids used in this study are listed in Table S1. The primers used in this study are listed in Table S2. Briefly, *S*. Tm strain ATCC 14028s served as the WT strain throughout this study. All bacterial strains were grown in LB broth containing 1% tryptone, 0.5% yeast extract, and 1% NaCl at 37°C but 30°C for strains carrying the temperature-sensitive plasmid PKD46. When needed, the arabinose and antibiotics were added to the medium at the following final concentrations: 100 mM arabinose, 100 μg/mL ampicillin, 25 μg/mL chloramphenicol, and 50 μg/mL kanamycin.

The mutant strain was generated using the λ-Red recombinase system as described previously.^46^ Briefly, the chloramphenicol resistance gene of pKD3 was amplified using primers with 38–40 bp of homology to the upstream and downstream regions flanking the mutant gene’s start and stop codons at their 5′ ends. The generated PCR products were electroporated into *S*. Tm strain ATCC 14028s carrying the temperature-sensitive plasmid PKD46 for homologous recombination. The mutant was selected based on its resistance to chloramphenicol and was further identified by PCR and DNA sequencing.

Complementation strains were generated by cloning the corresponding functional genes carrying the 3×FLAG tag into the pTrc99A plasmid and transforming the plasmid into the mutant strain. Strains carrying GFP were generated by transforming the pETDuet1-GFP plasmid into the corresponding strains. Eukaryotic expression vectors for colocalization by transfection were generated by cloning the corresponding functional genes and native promoters into the pCMV-C-GFP plasmid. A eukaryotic expression vector for immunoprecipitation was established by cloning the corresponding functional genes into the pcDNA3.1(+) plasmid. All resulting clones were verified by DNA sequencing.

### BMDM isolation and cell culture

BMDMs were isolated from female BALB/c mice as previously described.^47^ Briefly, 8-week-old mice were sacrificed, and femur and tibia bone marrow cells were flushed into RPMI 1640 medium (Gibco) supplemented with 10% fetal bovine serum (FBS; Gibco), passed through a 70 μm cell strainer to remove cell clumps, bone, hair, and other cells or tissues followed by centrifuging at 500 × g for 5 min. The collected cells were then suspended in RPMI 1640 medium containing 10% FBS, 1% penicillin/streptomycin, and 10 ng/mL monocyte-colony stimulating factor (M-CSF; MA5–23773; Invitrogen) and left to adhere at 37°C and 5% CO_2_. The medium was changed every 2 d, and BMDMs were used 7 d post-collection.

The RAW264.7 macrophage cell line was purchased from the Shanghai Institute of Biochemistry and Cell Biology of the Chinese Academy of Sciences (Shanghai, China) and cultured in RPMI 1640 medium supplemented with 10% FBS and incubated at 37°C with 5% CO_2_ in a humidified atmosphere for 24 h.

Before infection or transfection, BMDMs and RAW264.7 were grown to monolayers with 70–90% coverage in 12-well tissue culture plates with or without glass coverslips, 2-cm glass-bottom cell culture dishes, 10-cm cell culture dishes, or 75 cm^2^ culture bottles according to experimental requirements.

### The infection of BMDMs and intracellular replication assays

BMDMs were infected, as previously described, with slight modifications.^48^ Briefly, overnight-grown bacteria were inoculated into fresh LB medium at a ratio of 1:100 and further grown to the stationary phase until an OD_600_ of 1.5. Bacteria were pelleted and opsonized in the RPMI 1640 medium supplemented with 10% FBS for 20 min at 37°C. The bacterial culture was then added to BMDM monolayers at a multiplicity of infection (MOI) of 10. Subsequently, the plates were centrifuged at 1000 × g for 5 min to synchronize infection. After incubation for 40 min at 37°C with 5% CO_2_, the cell culture supernatant was discarded, and the infected BMDMs were washed thrice with phosphate-buffered saline (PBS) and supplemented with fresh RPMI 1640 medium containing 100 µg/mL gentamicin to kill the extracellular bacteria for 90 min. The infected BMDMs were washed thrice with PBS and incubated with 20 µg/mL gentamicin for the remaining infection time. For intracellular replication assays, BMDMs were seeded in 12-well tissue culture plates for 7 d before infection. At 2 and 8 h p.i., the supernatant was discarded, and the BMDMs were washed thrice with PBS and lysed with 0.1% Triton X-100. Lysates with serial gradient dilutions were plated on LB agar plates to count intracellular bacteria. Additionally, BMDMs were pretreated with the mitochondrial fragmentation inhibitor Mdivi1 (50 µM 475,856; Millipore) and mitochondrial fragmentation-inducing agent FCCP (10 µM, C2920; Sigma) 4 h before infection. BMDMs were then washed thrice with PBS. The intracellular fold replication was expressed as the bacterial burden recovered at 8 h p.i. relative to those at 2 h p.i. Triplicate wells were averaged for each data point, and three independent biological replicates were performed.

### Plasmid transfection

Eukaryotic expression vectors were extracted using the EndoFree Plasmid Midi Kit (CW2105S; CWBIO), according to the manufacturer’s instructions. Before transfection, RAW264.7 macrophages were seeded in 2-cm glass-bottom cell culture dishes or 10-cm cell culture dishes for 24 h. Then, RAW264.7 macrophages were transiently transfected with the indicated eukaryotic expression vectors using Lipofectamine LTX DNA Transfection Reagent (15338100; Invitrogen) according to the manufacturer’s instructions. Briefly, endofree plasmid DNA diluted in serum-free medium was mixed with PLUS Reagent. The diluted DNA solution was added to diluted Lipofectamine LTX Reagent and incubated for 5 min at room temperature. The resulting DNA – lipid complex was directly dropped into RAW264.7 and incubated for 24 h at 37°C with 5% CO_2_.

### Immunofluorescence

For the presentation of mitochondrial morphology and intracellular bacteria, BMDMs were seeded in 2-cm glass-bottom cell culture dishes for 7 d, followed by *S*. Tm infection and staining with 50 nM MitoTracker Deep Red FM (M22426; Invitrogen) according to the manufacturer’s instructions. For the colocalization of the indicated protein with mitochondria after transfection, RAW264.7 macrophages were stained with 50 nM MitoTracker Deep Red FM according to the manufacturer’s instructions. For the indicated protein colocalization with mitochondria after infection, BMDMs attached to glass coverslips were fixed with 4% paraformaldehyde for 10 min at room temperature, permeabilized, and blocked with 0.1% Triton X-100 in 5% bovine serum albumin (BSA) for 1 h at room temperature. Then, BMDMs were incubated with primary antibodies (Tomm20, 1:100, ab56783, Abcam; FLAG, 1:100, F1804, Sigma) overnight at 4°C, washed thrice with PBS, incubated with secondary antibodies (anti-mouse Alexa Fluor 594, 1:200, ab150116, Abcam; anti-rabbit Alexa Fluor 488, 1:200, ab150077, Abcam) for 1 h, and stained with DAPI for 5 min (C0065, Solarbio). After the final wash, the cells were covered with a mounting medium. Following staining, a confocal laser scanning microscope (Zeiss LSM800) was used to observe mitochondrial morphology, intracellular bacteria, the indicated protein colocalization with mitochondria, and image acquisition. For mitochondrial morphology, cells (20/field) of 15 random fields from three independent biological replicates were analyzed to determine the proportion of cells with fragmented mitochondria using ZEN 2.3 (blue edition). For intracellular bacteria, cells (20/field) of five random fields from three independent biological replicates were analyzed to determine the bacteria per BMDM using ZEN 2.3 (blue edition). For colocalization, 30 random fields from three independent biological replicates were analyzed the Pearson’s correlation coefficient with Fiji/ImageJ.

### Whole cell lysates after transfection

RAW264.7 macrophages were seeded in 10-cm cell culture dishes for 24 h. After transfection for 24 h, cells were washed thrice and lysed in RIPA buffer (R0010; Solarbio) containing 1 mM PMSF. Then, cell lysates were centrifuged at 15,000 × g for 10 min at 4°C, and the supernatants were collected and analyzed by immunoblotting.

### Mitochondrial isolation

Mitochondria were isolated as described previously.^49^ Briefly, BMDMs were seeded in 75-cm^2^ culture bottles for 7 d. After infection, BMDMs were collected at 800 × g for 5 min at 4°C, resuspended in ice-cold RSB Hypo buffer (10 mM NaCl, 1.5 mM MgCl_2_, and 10 mM Tris-HCl (pH7.5)) for 5–10 min, and homogenized to break the cells. Immediately, 2.5 × MS homogenization buffer (525 mM mannitol, 175 mM sucrose, 12.5 mM Tris-HCl (pH7.5), and 2.5 mM EDTA (pH7.5)) was added to the homogenate to acquire a final concentration of 1 × MS homogenization buffer. Then, the homogenate was centrifuged at 1300 × g for 5 min at 4°C, the supernatant was collected, and the centrifugation was repeated twice. The last collected supernatant was centrifuged at 15,000 × g for 15 min at 4°C to isolate the mitochondrial pellet. Finally, the mitochondrial pellet was lysed with 2% CHAPS in TBS buffer containing 1 mM PMSF, and the resulting lysate was quantified using a BCA Protein Assay Kit (C503021–0500; Sangon Biotech) according to the manufacturer’s instructions.

### Bacterial secretion assay

The detection of secreted SipA from *S*. Tm in LB culture supernatants was performed as previously described.^50^ Briefly, overnight bacterial cultures were subcultured in LB medium supplemented with 0.3 M NaCl under conditions that stimulated expression of the SPI1 T3SS at a ratio of 1:100 for 3 h at 37°C.^51^ Bacterial culture supernatants were separated by centrifugation at 12,000 × g for 10 min, filtered through a 0.22 µm syringe filter, and concentrated 100-fold with an Ultrafree centrifugal filter device (UFC9030; Millipore) with a 30-kDa cutoff at 4°C followed by quantification through sodium dodecyl sulfate-polyacrylamide gel electrophoresis (SDS-PAGE) and Coomassie blue staining. The separated bacterial cells were resuspended in PBS containing 1 mM PMSF (B111–01; GenStar) and disrupted by sonication at 4°C. Bacterial culture supernatants and bacterial cell lysates were further analyzed by immunoblotting.

Additionally, the detection of secreted SipA in *S*. Tm-infected BMDMs was conducted as previously described.^52^ BMDMs were seeded in 10-cm cell culture dishes for 7 d. After 8 h infection, BMDMs were washed thrice with PBS and then treated with 30 µg/mL of proteinase K (AA1907; SparkJade) in PBS for 15 min at 37°C in a CO_2_ incubator to eliminate cell-surface-associated SipA proteins. Cells detached during the proteinase treatment and were subsequently collected by centrifugation at 600 × g for 5 min and lysed in PBS containing 0.1% Triton X-100 and 1 mM PMSF. The cell lysates were centrifuged at 15,000 × g for 10 min at 4°C and the separated supernatants were filtered through a 0.22 µm syringe filter and proteins were analyzed by immunoblotting.

### MtROS determination

MtROS were detected with a mitochondrial ROS detector, MitoSOX Red (M36008; Invitrogen), using the confocal laser scanning microscope. Briefly, BMDMs were seeded in 2-cm glass-bottom cell culture dishes for 7 d. After infection, BMDMs were treated with 5 µM MitoSOX for 10 min at 37°C, washed thrice with PBS, and stained with DAPI for 5 min. After washing thrice with PBS, the confocal laser scanning microscope was used to observe mtROS fluorescence and acquire images. Cells (20/field) of 15 random fields from three independent biological replicates were quantified for mtROS fluorescence intensity using ZEN 2.3 (blue edition).

### Measurement of ΔΨ_m_

The ΔΨ_m_ was measured with TMRM (I34361; Invitrogen) using the confocal laser scanning microscope. Briefly, BMDMs were seeded in 2-cm glass-bottom cell culture dishes for 7 d. After infection, BMDMs were loaded with 100 nM TMRM for 30 min at 37°C and washed thrice with PBS. Confocal laser scanning microscope was used for ΔΨ_m_ fluorescence observation and image acquisition. Cells (20/field) of 15 random fields from three independent biological replicates were quantified for ΔΨ_m_ fluorescence intensity using ZEN 2.3 (blue edition).

### MtDNA extraction and quantification

Released mtDNA was extracted from the infected BMDMs using the Digitonin method.^53^ Briefly, BMDMs were seeded in 75-cm^2^ culture bottles for 7 d. After infection, the BMDMs were collected by centrifugation at 1000 × g for 3 min. Subsequently, the cell pellet was resuspended in digitonin buffer (150 mM NaCl, 50 mM HEPES, and 25 µg/mL digitonin). The cell suspension was rotated for 10 min and centrifugated at 1000 × g for 3 min. The supernatant was centrifuged again at 16,000 × g for 25 min to obtain a new supernatant containing cytosolic mtDNA (cmtDNA) released from the mitochondria. The remaining pellet containing the nuclear genome was prepared for nuclear DNA (nucDNA) acquisition using the SPARKeasy Tissue/Cell DNA Kit (AA1002-B; SparkJade). The purity ratios (A260/280 and A260/230) and DNA yield (nanograms per microliter) of both nucDNA and cmtDNA were determined with the NanoDrop 2000 spectrophotometer (Thermo Fisher Scientific, Waltham, MA, USA).

The mtDNA copy numbers were determined by qPCR performed on ABI 7500 thermocycler sequence detection (Applied Biosystems) using SYBR green fluorescence dye. Two mitochondrial (*16S* and *ND4*) and one nuclear (*PMP2*2) genes were amplified.^54^ The qPCR primers used in this study are listed in Table S2. The *PMP22* gene was used as a reference control, and the relative expression level of candidate targets was calculated as fold-change values using the 2^−ΔΔCT^ method. The mtDNA copy numbers acquired by cmtDNA/nucDNA were standardized to the control. The data were collected from three biological replicates.

### Cytochrome c release

Cytochrome *c* release was measured using a cytochrome *c* Releasing Apoptosis Assay kit (ab65311; Abcam) according to the manufacturer’s manual. Briefly, BMDMs were seeded in 75-cm^2^ culture bottles for 7 d. After infection, BMDMs were collected and centrifuged at 600 × g for 5 min at 4°C. Subsequently, the cells were homogenized and separated into the cytosolic and mitochondrial fractions by centrifugation. The cytosolic fraction contained the released cytochrome *c* from the mitochondria, and the mitochondrial fraction contained the remaining cytochrome *c*. To release the remaining cytochrome *c* from the mitochondria, the mitochondrial fraction was lysed with 2% CHAPS in TBS buffer containing 1 mM PMSF. Finally, the cytosolic and mitochondrial fractions were quantified using a BCA Protein Assay Kit (C503021–0500; Sangon Biotech) according to the manufacturer’s instructions.

### Immunoblotting

Protein samples were boiled in 1 × SDS loading buffer and separated using a 4–12% gradient Bis-Tris Gel (M00653; GenScript) by SDS-PAGE. Subsequently, the separated proteins were transferred onto a 0.45 µm PVDF membrane (IPVH00010; Millipore) and blocked with 5% skimmed milk at room temperature for 2 h. Then, the blots were incubated for another 2 h at room temperature with the following primary antibodies: anti-FLAG (1:1000, F1804; Sigma), anti-GFP (1:1000, K200047M; Solarbio), anti-cytochrome *c* (1:1000, MA5–11674; Invitrogen), anti-Drp1 (1:1000, ab184247; Abcam), anti-SipA (Shanghai Willget Biotech Co., Ltd.), anti-GroEL (1:1000, ab82592; Abcam), anti-Tomm20 (1:1000, ab56783; Abcam), and anti-β-Actin (1:1000, CW0096; CWBIO). To detect proteins, the blots were incubated with the corresponding horseradish peroxidase (HRP)-conjugated anti-rabbit (EF0002; SparkJade) or anti-mouse (EF0001; SparkJade) secondary antibodies at room temperature for 1 h. Finally, the blots were visualized with an ECL detection kit (D601039; Sangon Biotech) using an Amersham^TM^ Imager 600 system (General Electric). Immunoblotting bands were quantified using the ImageJ software. The data were collected from three biological replicates.

### Mice infection

Mouse infection assays were performed as previously described with slight modifications.^46^ Briefly, the overnight-grown bacteria were subcultured as 1:100 in fresh LB medium and grown to the stationary phase. Subsequently, the bacteria were collected and washed thrice with sterile 0.9% NaCl and serially diluted to 1 × 10^7^ CFU/mL in 0.9% NaCl for intraperitoneal infection. Female BALB/c mice were injected intraperitoneally with 1 × 10^6^ CFU in 0.1 mL sterile 0.9% NaCl. The infected mice were euthanized at 3 d p.i. The spleen and liver were harvested, homogenized, serially diluted in PBS, and plated on LB agar plates to count the bacterial CFU. Experiments were performed with three independent biological replicates, where each experiment employed four mice, and the data were pooled (*n* = 12).

### Growth assay

To determine the growth curve of each WT, Δ*sipA*, Δ*sipA+*pFSipA, and Δ*sipA+*pMSipA strains, overnight-grown bacteria were adjusted to an OD_600_ of 1.5 and diluted 1:1000 in LB and RPMI 1640 medium. Next, a 200 μL aliquot was added to a 96-well microplate and incubated at 37°C with shaking at 180 rpm for 24 h. Absorbance at 600 nm was recorded using a programmed procedure. The experiment was independently performed in triplicate.

### Immunoprecipitation

RAW264.7 macrophages were seeded in 10-cm cell culture dishes for 24 h and transfected with the endofree plasmid pcDNA3.1(+) and pcDNA3.1(+)-SipA. After 24 h, the RAW264.7 macrophages were subjected to immunoprecipitation. Briefly, the cells were washed thrice and lysed in 1 mL RIPA buffer containing a complete protease inhibitor cocktail. Lysates were centrifuged at 15,000 × g for 10 min at 4°C, and the supernatants were collected. Subsequently, 50 µL Dynabeads (10001D; Novex) were incubated with anti-Drp1 antibody (ab184247; Abcam) for 30 min at room temperature on a rotation shaker followed by washing with PBST (PBS (pH 7.4) with 0.02% Tween-20). The Dynabeads – antibody complex was incubated with supernatant lysates overnight at 4°C on a rotatory shaker and washed thrice with PBST. Finally, the proteins bound to the beads were eluted with an elution buffer (50 mM glycine, pH 2.8) and boiled in SDS loading buffer. The protein samples were resolved in 4–12% gradient Bis-Tris Gel and analyzed by immunoblotting with anti-Drp1 and anti-SipA antibodies. Experiments were performed with three independent biological replicates.

### Statistical analyses

Statistical significance was analyzed with GraphPad Prism 8.0.1 software (GraphPad Inc., San Diego, CA, USA) using the two-way ANOVA, two-tailed unpaired Student’s *t*-test and Mann–Whitney U-test according to the test requirements (as stated in the figure legends). *P*-values <0.05, 0.01, and 0.001 were considered statistically significant (*), highly significant (**), and extremely significant (***), respectively, and ns represents no significant difference.

## Supplementary Material

Supplementary material revised clean.docx

## Data Availability

The authors confirm that the data supporting the findings of this study are available within the article [and/or] its supplementary materials.
