# Supplementary material for: Salmonella enterica serovar Typhimurium remodels mitochondrial dynamics of macrophages via the T3SS effector SipA to promote intracellular proliferation
Source: Gut Microbes. 2024 Feb 14;16(1):2316932. doi: 10.1080/19490976.2024.2316932 (PMC10877990; doi:10.1080/19490976.2024.2316932)
Supplement: Supplementary material revised clean.docx [file KGMI_A_2316932_SM5109.docx]

**Supplemental Materials for**

***Salmonella enterica* serovar Typhimurium remodels mitochondrial dynamics of macrophages via the T3SS effector SipA to promote intracellular proliferation**

Xingmei Liu^a,b,†^, Yutao Liu^a,b,†^, Xinyu Zhao^a,b^, Xueping Li^a,b^, Ting Yao^a,b^, Ruiying Liu^a,b^, Qian Wang^a,b^, Qiushi Wang^a,b^, Dan Li^a,b^, Xintong Chen^a,b^, Bin Liu^a,b,c,*^, and Lu Feng ^a,b,*^

*^a^National Key Laboratory of Intelligent Tracking and Forecasting for Infectious Diseases, TEDA Institute of Biological Sciences and Biotechnology, Nankai University, Tianjin, China.*

*^b^Key Laboratory of Molecular Microbiology and Technology, Nankai University, Ministry of Education, Tianjin, China.*

*^c^Nankai International Advanced Research Institute, Nankai University Shenzhen, China*

^†^ These authors contributed equally to this work.

*Corresponding authors. Email: fenglu63@nankai.edu.cn (Lu Feng); liubin1981@nankai.edu.cn (Bin Liu).

**This file includes:**

**Figures S1 to S5**

**Tables S1 to S2**

**Supplementary Figures**


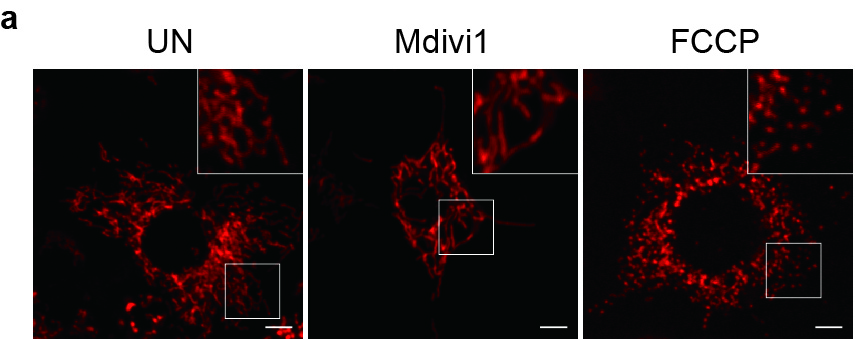


**Figure S1. Effects of different reagents on mitochondrial morphology. (a)** Representative immunofluorescence images of mitochondrial morphology in BMDMs treated with Mdivi1 or FCCP at 4 h. Mitochondria, red. UN, untreated cells. Scale bars, 5 µm.


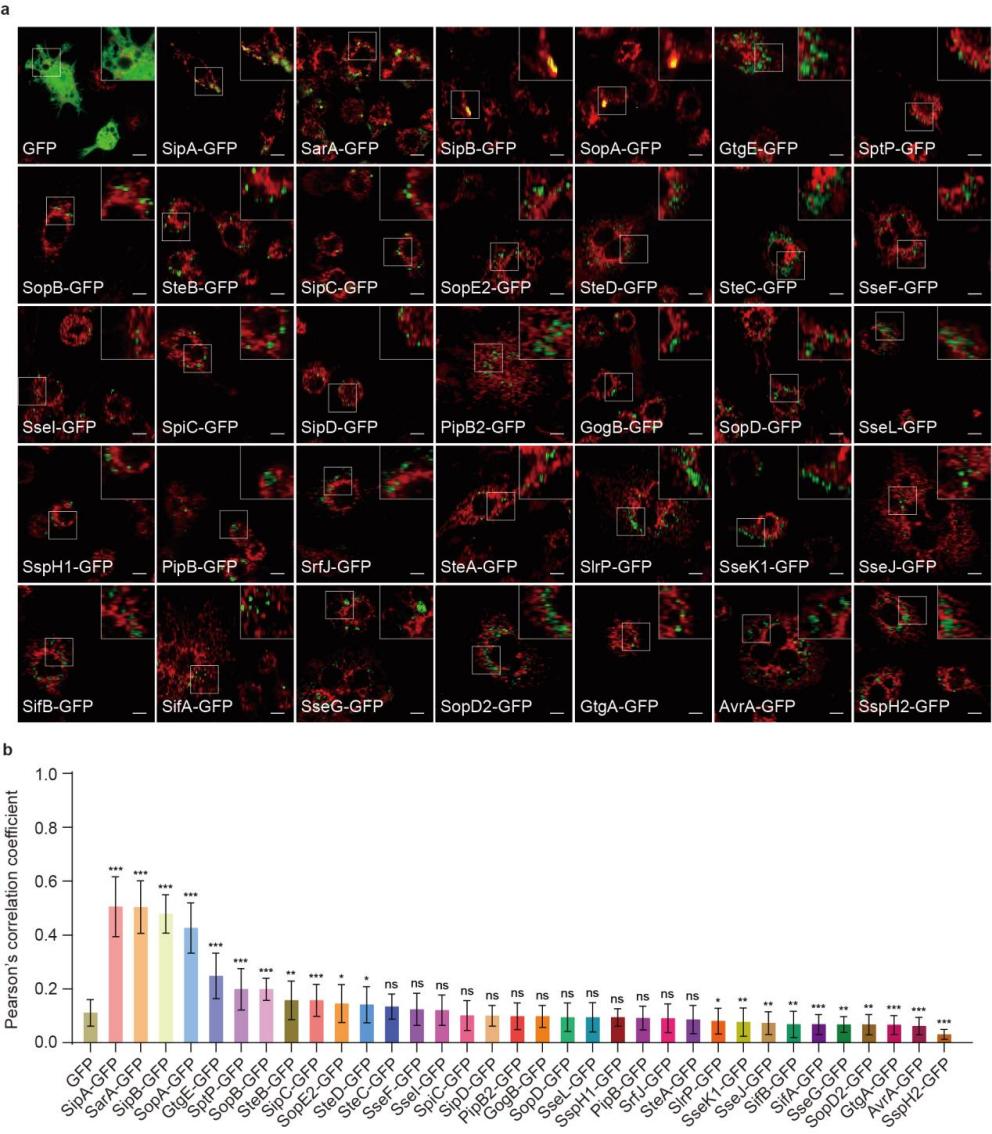


**Figure S2. Identification of 34 effectors colocalizing with the mitochondria. (a)** Representative immunofluorescence images of Raw264.7 macrophages transfected with eukaryotic expression vectors tagged with GFP of the 34 effectors during 24 h. Mitochondria, red; the indicated effectors, green. Scale bars, 10 µm. **(b)** Pearson’s correlation coefficient for the indicated effectors and mitochondria colocalization after 24 h of transfection. GFP served as the control. Data are presented as mean ± SD (n = 30 fields). Significant differences were assessed using two-way ANOVA (b). Error bars represent SD. **P* < 0.05, ***P* < 0.01, ****P* < 0.001; ns, no significant difference.


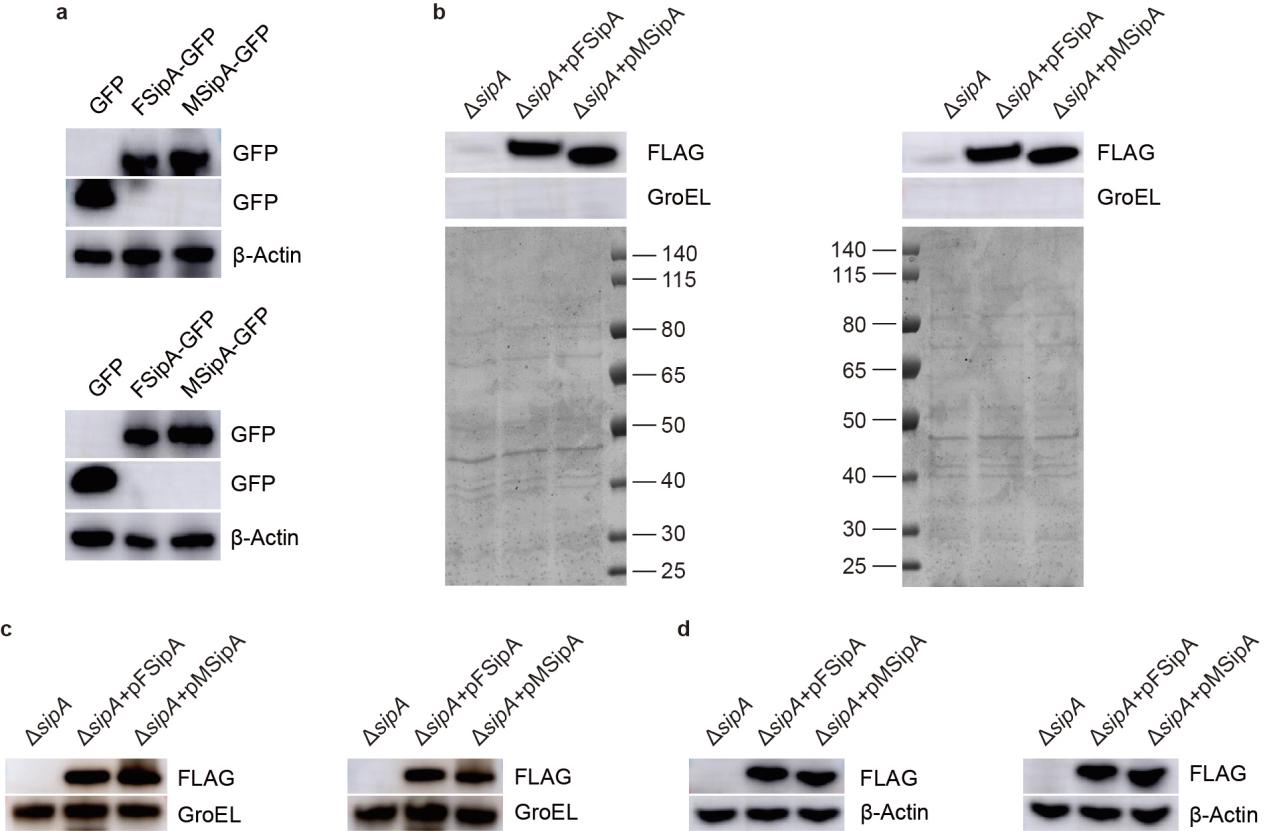


**Figure S3. Repeated experiments for secretion of SipA independent of the N-terminal MTS. (a)** Expression levels of SipA in Raw264.7 macrophages transfected with pCMV-C-GFP, pCMV-FSipA-GFP, or pCMV-MSipA-GFP plasmids for 24 h followed by immunobloting with antibody against GFP tag. **(b)** Detection of SipA secreted by Δ*sipA*, Δ*sipA+*pFSipA and Δ*sipA+*pMSipA under T3SS inducing conditions in bacterial culture supernatants. Total proteins were quantified by SDS-PAGE stained with Coomassie blue staining. Protein SipA was detected by immunoblotting using antibody against the FLAG tag. **(c)** Detection of SipA in bacterial cell lysates from Δ*sipA*, Δ*sipA+*pFSipA and Δ*sipA+*pMSipA by immunoblotting using antibody against the FLAG tag. **(d)** Detection of SipA secreted in BMDMs infected with Δ*sipA*, Δ*sipA+*pFSipA and Δ*sipA+*pMSipA by immunoblotting with an antibody against the FLAG tag. FSipA, full-length SipA; MSipA, MTS-deleted SipA. β-Actin, cytosolic control; GroEL, bacterial control.


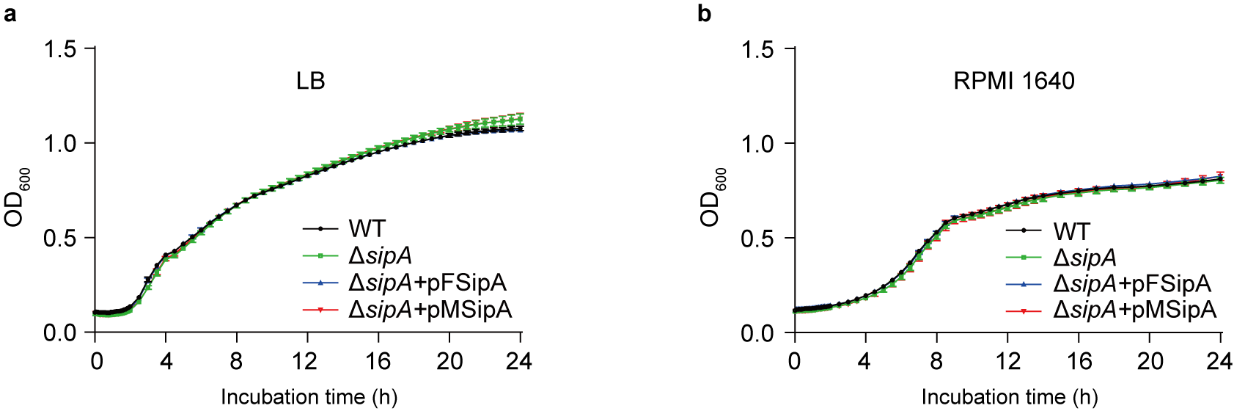


**Figure S4. (a**, **b) Growth curves of WT, Δ*sipA*, Δ*sipA+*pFSipA, and Δ*sipA+*pMSipA strains in LB and RPMI1640 medium.** Data are presented as mean ± SD (n = 3 independent experiments). Error bars represent SD.


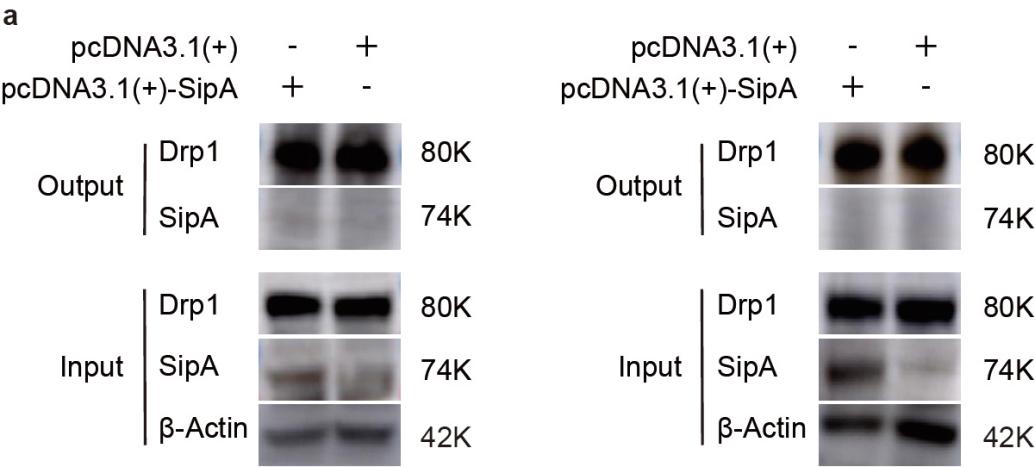


**Figure S5. SipA indirectly interact with Drp1.** **(a)** Repeated experiments for immunoprecipitation of SipA and Drp1 in Raw264.7 macrophages. β-Actin, cytosolic control.

**Supplementary Tables**

**Table S1. Strains and plasmids used in this study**

| **Strains** | **Genetype or description** | **Source** |
| --- | --- | --- |
| WT | Wild-type *Salmonella enterica* subsp. enterica serovar Typhimurium ATCC 14028s | ATCC* |
| WT+GFP | WT harboring the plasmid pETDuet1-GFP; Ap^R^ | This research |
| Δ*sipA* | *sipA* deletion mutant in WT; Cm^R^ | This research |
| Δ*sipA*+GFP | Δ*sipA* harboring the plasmid pETDuet1-GFP; Cm^R^, Ap^R^ | This research |
| Δ*sipA+*pFSipA | Δ*sipA* harboring the plasmid pTr-FSipA-FLAG; Cm^R^, Ap^R^ | This research |
| Δ*sipA+*pFSipA+GFP | Δ*sipA* harboring the two plasmids pTr-FSipA-FLAG and pETDuet1-GFP; Cm^R^, Ap^R^ | This research |
| Δ*sipA+*pMSipA | Δ*sipA* harboring the plasmid pTr-MSipA-FLAG; Cm^R^, Ap^R^ | This research |
| Δ*sipA+*pMSipA+GFP | Δ*sipA* harboring the two plasmids pTr-MSipA-FLAG and pETDuet1-GFP; Cm^R^, Ap^R^ | This research |
| DH5α | *E. Coli* DH5α/λpir strain for gene cloning | Lab collection |
| **Plasmids** | | |
| pKD3 | Carrying the chloramphenicol resistance gene; Cm^R^ | Lab collection |
| pKD46 | Red recombinase plasmid; Ap^R^ | Lab collection |
| pTrc99A | Prokaryotic complemented vector; Ap^R^ | Lab collection |
| pETDuet1-GFP | pETDuet1 carrying the GFP; Ap^R^ | Lab collection |
| pCMV-C-GFP | Eukaryotic expression vector for expression of GFP; Km^R^ | Lab collection |
| pcDNA3.1(+) | Eukaryotic expression vector; Ap^R^ | Lab collection |
| pTr-FSipA-FLAG | pTrc99A carrying the FSipA-FLAG fusion fragment; Ap^R^ | This research |
| pTr-MSipA-FLAG | pTrc99A carrying the MSipA-FLAG fusion fragment; Ap^R^ | This research |
| pCMV-FSipA | pCMV-C-GFP carrying the FSipA and its native promotor region; Km^R^ | This research |
| pCMV-MSipA | pCMV-C-GFP carrying the MSipA and its native promotor region; Km^R^ | This research |
| pCMV-SopA | pCMV-C-GFP carrying the SopA and its native promotor region; Km^R^ | This research |
| pCMV-SopE2 | pCMV-C-GFP carrying the SopE2 and its native promotor region; Km^R^ | This research |
| pCMV-AvrA | pCMV-C-GFP carrying the AvrA and its native promotor region; Km^R^ | This research |
| pCMV-SipC | pCMV-C-GFP carrying the SipC and its native promotor region; Km^R^ | This research |
| pCMV-SipB | pCMV-C-GFP carrying the SipB and its native promotor region; Km^R^ | This research |
| pCMV-SipD | pCMV-C-GFP carrying the SipD and its native promotor region; Km^R^ | This research |
| pCMV-SopB | pCMV-C-GFP carrying the SopB and its native promotor region; Km^R^ | This research |
| pCMV-SptP | pCMV-C-GFP carrying the SptP and its native promotor region; Km^R^ | This research |
| pCMV-SopD | pCMV-C-GFP carrying the SopD and its native promotor region; Km^R^ | This research |
| pCMV-SopE | pCMV-C-GFP carrying the SopE and its native promotor region; Km^R^ | This research |
| pCMV-SifB | pCMV-C-GFP carrying the SifB and its native promotor region; Km^R^ | This research |
| pCMV-SseK2 | pCMV-C-GFP carrying the SseK2 and its native promotor region; Km^R^ | This research |
| pCMV-SpvB | pCMV-C-GFP carrying the SpvB and its native promotor region; Km^R^ | This research |
| pCMV-SseC | pCMV-C-GFP carrying the SseC and its native promotor region; Km^R^ | This research |
| pCMV-SrfJ | pCMV-C-GFP carrying the SrfJ and its native promotor region; Km^R^ | This research |
| pCMV-SseF | pCMV-C-GFP carrying the SseF and its native promotor region; Km^R^ | This research |
| pCMV-SseG | pCMV-C-GFP carrying the SseG and its native promotor region; Km^R^ | This research |
| pCMV-SteD | pCMV-C-GFP carrying the SteD and its native promotor region; Km^R^ | This research |
| pCMV-GogB | pCMV-C-GFP carrying the GogB and its native promotor region; Km^R^ | This research |
| pCMV-SseK1 | pCMV-C-GFP carrying the SseK1 and its native promotor region; Km^R^ | This research |
| pCMV-SteC | pCMV-C-GFP carrying the SteC and its native promotor region; Km^R^ | This research |
| pCMV-PipB | pCMV-C-GFP carrying the PipB and its native promotor region; Km^R^ | This research |
| pCMV-PipB2 | pCMV-C-GFP carrying the PipB2 and its native promotor region; Km^R^ | This research |
| pCMV-SifA | pCMV-C-GFP carrying the SifA and its native promotor region; Km^R^ | This research |
| pCMV-SopD2 | pCMV-C-GFP carrying the SopD2 and its native promotor region; Km^R^ | This research |
| pCMV-SpiC | pCMV-C-GFP carrying the SpiC and its native promotor region; Km^R^ | This research |
| pCMV-SseI | pCMV-C-GFP carrying the SseI and its native promotor region; Km^R^ | This research |
| pCMV-SseJ | pCMV-C-GFP carrying the SseJ and its native promotor region; Km^R^ | This research |
| pCMV-SseL | pCMV-C-GFP carrying the SseL and its native promotor region; Km^R^ | This research |
| pCMV-SspH2 | pCMV-C-GFP carrying the SspH2 and its native promotor region; Km^R^ | This research |
| pCMV-GtgA | pCMV-C-GFP carrying the GtgA and its native promotor region; Km^R^ | This research |
| pCMV-SsrA | pCMV-C-GFP carrying the SsrA and its native promotor region; Km^R^ | This research |
| pCMV-SseB | pCMV-C-GFP carrying the SseB and its native promotor region; Km^R^ | This research |
| pCMV-SseD | pCMV-C-GFP carrying the SseD and its native promotor region; Km^R^ | This research |
| pCMV-SsaJ | pCMV-C-GFP carrying the SsaJ and its native promotor region; Km^R^ | This research |
| pCMV-SsaV | pCMV-C-GFP carrying the SsaV and its native promotor region; Km^R^ | This research |
| pCMV-SlrP | pCMV-C-GFP carrying the SlrP and its native promotor region; Km^R^ | This research |
| pCMV-SteB | pCMV-C-GFP carrying the SteB and its native promotor region; Km^R^ | This research |
| pCMV-SarA | pCMV-C-GFP carrying the SarA and its native promotor region; Km^R^ | This research |
| pCMV-GtgE | pCMV-C-GFP carrying the GtgE and its native promotor region; Km^R^ | This research |
| pCMV-SpvD | pCMV-C-GFP carrying the SpvD and its native promotor region; Km^R^ | This research |
| pCMV-PipA | pCMV-C-GFP carrying the PipA and its native promotor region; Km^R^ | This research |
| pCMV-SteA | pCMV-C-GFP carrying the SteA and its native promotor region; Km^R^ | This research |
| pCMV-SpvC | pCMV-C-GFP carrying the SpvC and its native promotor region; Km^R^ | This research |
| pCMV-SspH1 | pCMV-C-GFP carrying the SspH1 and its native promotor region; Km^R^ | This research |
| pCMV-GogA | pCMV-C-GFP carrying the GogA and its native promotor region; Km^R^ | This research |
| pcDNA3.1(+)-SipA | pcDNA3.1(+) carrying the SipA; Ap^R^ | This research |

^*^, ATCC, American Type Culture Collection, Manassas, Virginia, USA.

**Table S2. Primers used in this study (5'-3').**

| Primers for gene mutation | | |
| --- | --- | --- |
| *sipA* | F | AAAAGCTTCCTGCAAGGATAACAGAAGAGGATATTAATAGTGTAGGCTGGAGCTGCTTCG |
| *sipA* | R | TGCTTCAATATCCATATTCATCGCATCTTTCCCGGTTAACATATGAATATCCTCCTTAG |
| Primers for complement construction | | |
| F*sipA* | F | CCGGAATTCATGGTTACAAGTGTAAGGACTCAG |
| F*sipA* | R | CTAGTCTAGATTACTATTTATCGTCGTCATCTTTGTAGTCGATATCATGATCTTTATAATCACCGTCATGGTCTTTGTAGTCACGCTGCATGTGCAAGCCATCAAC |
| M*sipA* | F | CCGGAATTCatgagtgccacagcgacgctgtcaggggaaat |
| M*sipA* | R | CTAGTCTAGATTACTATTTATCGTCGTCATCTTTGTAGTCGATATCATGATCTTTATAATCACCGTCATGGTCTTTGTAGTCACGCTGCATGTGCAAGCCATCAAC |
| Primers for colocalization of transfection | | |
| F*sipA* | F | ggaattcgttatgtcgtcaccgttgatctga |
| F*sipA* | R | gctctagaacgctgcatgtgcaagccatc |
| M*sipA* | P1 | ggaattcgttatgtcgtcaccgttgatctga |
| M*sipA* | P2 | atttcccctgacagcgtcgctgtggcactcattattaatatcctcttctgttatcctt |
| M*sipA* | P3 | aaggataacagaagaggatattaataatgagtgccacagcgacgctgtcaggggaaat |
| M*sipA* | P4 | gctctagaacgctgcatgtgcaagccatc |
| *sopA* | F | ATAAGAATGCGGCCGCGTCCGCCGACGATATCCCGA |
| *sopA* | R | CTAGTCTAGACGCCCAGGCCAGTGGCAGGAT |
| *sopE2* | F | CGGGATCCAATCGTAACAACATCAGCA |
| *sopE2* | R | CTAGTCTAGAGGAGGCATTCTGAAGATAC |
| *avrA* | F | CGGGATCCGTTTGGGGATGGACTCTTCACC |
| *avrA* | R | CTAGTCTAGACGGTTTAAGTAAAGACTTA |
| *sipC* | F | CGGGATCCAAAGTGGCGTTGGGCATGGAA |
| *sipC* | R | CTAGTCTAGAAGCGCGAATATTGCCTGCGAT |
| *sipB* | F | ATAAGAATGCGGCCGCTTTTTACAATCCCGATTACAC |
| *sipB* | R | CTAGTCTAGATGCGCGACTCTGGCGCAGAATAA |
| *sipD* | F | CCGGAATTCCGGTCGGTGGTATTGCA |
| *sipD* | R | CTAGTCTAGATCCTTGCAGGAAGCTTTTGGCG |
| *sopB* | F | ATAAGAATGCGGCCGCCGTTACTTTACGCAGGAGTA |
| *sopB* | R | CTAGTCTAGAAGATGTGATTAATGAAGAAATGCC |
| *sptP* | F | CGGGATCCGCTTATTATTACTCGATAGCG |
| *sptP* | R | CTAGTCTAGAGCTTGCCGTCGTCATAAGCAA |
| *sopD* | F | ATAAGAATGCGGCCGCCCACGAATAATTATTATAAAT |
| *sopD* | R | CTAGTCTAGATGTCAGTAATATATTACGAC |
| *sopE* | F | CCGGAATTCAATCGTAACAACATCAGCA |
| *sopE* | R | CTAGTCTAGAGGAGGCATTCTGAAGATACT |
| *sifB* | F | CCGGAATTCCTTTCTCTAAAAATAATATAG |
| *sifB* | R | CTAGTCTAGAACTCTGGTGATGAGCCTCA |
| *sseK2* | F | CGGGATCCCCTTACGCAGGCTTATTTTTTGCG |
| *sseK2* | R | CTAGTCTAGACCTCCAAGAACTGGCAGTT |
| *spvB* | F | CCGGAATTCAAACCGCCGACTATACGCCA |
| *spvB* | R | CTAGTCTAGATGAGTTGAGTACCCTCATG |
| *sseC* | F | CGGGATCCATTGCTGACGATATATGATGCC |
| *sseC* | R | CTAGTCTAGA AGCGCGATAGCCAGCTATTCT |
| *srfJ* | F | CCGGAATTCGACTGGAAACAGCGCTTTATTG |
| *srfJ* | R | CTAGTCTAGAGATCGACTCCTGCCGCCATA |
| *sseF* | F | CCGGAATTCGTGATGGCCCAGCCATGGA |
| *sseF* | R | CTAGTCTAGATGGTTCTCCCCGAGATGTATG |
| *sseG* | F | CCGGAATTCACGTTCAGGAATCGCTATTTC |
| *sseG* | R | CTAGTCTAGACTCCGGCGCACGTTGTTCT |
| *steD* | F | CCGGAATTCTGATAACGAAACGGAGAA |
| *steD* | R | CTAGTCTAGATGGCCAGGCTGGCCGGGTTCTGA |
| *gogB* | F | CCGGAATTCACTGTAGCTTTGAAATCGTC |
| *gogB* | R | CTAGTCTAGAACGATTTCTATTTTTAGGC |
| *sseK1* | F | CCGGAATTCTAAACCTGTTTTATTGATTG |
| *sseK1* | R | CTAGTCTAGACTGCACATGCCTCGCCCAT |
| *steC* | F | CCGGAATTCGGATAGCAAGTACGATAGCGA |
| *steC* | R | CTAGTCTAGATTTTTTTAATTCATCCTTT |
| *pipB* | F | CCGGAATTCTTAAGTAAATTTTCGCTCAAC |
| *pipB* | R | CTAGTCTAGAAAATATCGGATGGGGGAAAA |
| *pipB2* | F | CCGGAATTCATGATGTTATATACTCTAA |
| *pipB2* | R | CTAGTCTAGAAATATTTTCACTATAAAATTCGT |
| *sifA* | F | CCGGAATTCTCCAGGCATGAAGTTTATTC |
| *sifA* | R | CTAGTCTAGATAAAAAACAACATAAACAGCCGC |
| *sopD2* | F | CCG GAATTC AAGTGTATCATCCATAGCGCC |
| *sopD2* | R | CTAGTCTAGA AAGCATATTGCGACAACTCGA |
| *spiC* | F | CCGGAATTCGGAAGATAAATTTCTTCATA |
| *spiC* | R | CTAGTCTAGATACCCCACCCGAATAAAGT |
| *sseI* | F | CCGGAATTCTTATCTGAATCGTTAAGT |
| *sseI* | R | CTAGTCTAGACATTTTACCTATTAAGGAA |
| *sseJ* | F | CCGGAATTCGCTCATACTCACGCCAGCACA |
| *sseJ* | R | CTAGTCTAGATTCAGTGGAATAATGATGAGC |
| *sseL* | F | CCGGAATTCGATAACGACGTTACTGTT |
| *sseL* | R | CTAGTCTAGACTGGAGACTGTATTCATA |
| *sspH2* | F | CCGGAATTCAAAGGGTTTATTCGCCGGAAG |
| *sspH2* | R | CTAGTCTAGAGTTACGACGCCACTGAACGTT |
| *gtgA* | F | CCCAAGCTTAGAATTCAGGAGTCTTGTCA |
| *gtgA* | R | CTAGTCTAGAATTACTAAATTCGTAGGCGA |
| *ssrA* | F | CCG GAATTCTCTACATATACCTTGTCACAGGCG |
| *ssrA* | R | CTAGTCTAGA AGTAATGGTGTAGTTTTTG |
| *sseB* | F | CGGGATCCTGAACAACACCGGCAGCG |
| *sseB* | R | CTAGTCTAGAGATGATGCGGTTCTGTTT |
| *sseD* | F | CGGGATCCTTTAAAGGCTTACAGGCTATCAG |
| *sseD* | R | CTAGTCTAGACCTCGTTAATGCCCGGAGTATT |
| *ssaJ* | F | CGGGATCCTGCGTCTGTTATTTCCTGCA |
| *ssaJ* | R | CTAGTCTAGA AAAACGGCGTCTCAGGCAAAAAT |
| *ssaV* | F | CGGGATCCAGGATTAGCTGAACGGCC |
| *ssaV* | R | CTAGTCTAGATTCTTCATTGTCCGCCAACTC |
| *slrP* | F | CCGGAATTCTGAACCCAACGCTAATTTTCC |
| *slrP* | R | CTAGTCTAGATCGCCAGTAGGCGCTCATGAG |
| *steB* | F | CCGGAATTCACAGAGGTAATGCCGGTAA |
| *steB* | R | CTAGTCTAGATCTGACATTACCATTTGAGTG |
| *sarA* | F | CCGGAATTCCCCCACCAGATAATACAAAACA |
| *sarA* | R | CTAGTCTAGATTCATCCGGGAAAACCTCTGC |
| *gtgE* | F | CCGGAATTCAAAAGCGATCGCGAAAAT |
| *gtgE* | R | CTAGTCTAGATAAAATGGTACACCAGTC |
| *spvD* | F | CGGGATCCTTTACGTGAGGAACCGTTT |
| *spvD* | R | CTAGTCTAGAATCGTGTTTTTCATCATAAGCCC |
| *pipA* | F | CGGGATCCTTCAGCGTATTACTTGTTGGC |
| *pipA* | R | CTAGTCTAGATTTATTGAAGATGTAGACC |
| *steA* | F | CCCAAGCTTATGTGGTGCGGTGTGGTCA |
| *steA* | R | CTAGTCTAGAATAATTGTCCAAATAGTTATG |
| *spvC* | F | CGGGATCCTGAGGGTACTCAACTCAT |
| *spvC* | R | CTAGTCTAGACTCTGTCATCAAACGATAAAACGG |
| *sspH1* | F | ATAAGAATGCGGCCGCGATGAATGGCTGGTTCACTAT |
| *sspH1* | R | CTAGTCTAGAGTTAAGACGCCACCGGGCTGT |
| *gogA* | F | CGGGATCCAGAATTCAGGAGTCTTGTC |
| *gogA* | R | CTAGTCTAGAATTACTAGATTCGTAGGCGA |
| Primers for IP | | |
| *sipA* | F | CGGGATCCATGGTTACAAGTGTAAGGACTC |
| *sipA* | R | GCTCTAGATTAACGCTGCATGTGCAAGCCAT |
| Primers for qPCR | | |
| cmtDNA-*16S* | F | CGTCTATGTGGCAAAATAGTGAGAA |
| cmtDNA-*16S* | R | CCAGCTATCACCAAGCTCGTT |
| cmtDNA-*ND4* | F | CACATGGCCTCACATCATCAC |
| cmtDNA-*ND4* | R | GTGGATCCGTTCGTAGTTGGA |
| nucDNA-*PMP22* | F | TTCGTCAGTCCCACAGTTTTCTC |
| nucDNA-*PMP22* | R | ACTCGCTAGTCCCAAGGGTCTA |
